# Growth estimation of the larger foraminifer *Heterostegina depressa* by means of population dynamics

**DOI:** 10.7717/peerj.6096

**Published:** 2019-01-16

**Authors:** Wolfgang Eder, Julia Woeger, Shunichi Kinoshita, Johann Hohenegger, Antonino Briguglio

**Affiliations:** 1 Department of Palaeontology, University of Vienna, Vienna, Austria; 2 Dipartimento di Scienze della terra, dell’ambiente e della vita (DISTAV), Università degli Studi di Genova, Genova, Italy

**Keywords:** Growth estimation, Chamber building rate, Natural laboratory, Carbonate producer

## Abstract

In* Heterostegina depressa*, the flagship species of laboratory investigations of larger benthic foraminifera (LBF) since the 70’s, the timing of reproduction, longevity and natural chamber building rates are still understudied. A recently developed method, the natural laboratory (sensu Hohenegger), has been applied on *H. depressa* populations from Sesoko Jima, NW Okinawa, Japan. An averaged chamber building rate and longevity of *H. depressa* were calculated based on 17 monthly samplings at fixed stations. All samples were collected at 20 and 50 m water depths using SCUBA. Live populations were dried and investigated by microCT. The monthly frequency distributions of chamber numbers and test diameters have been decomposed in normally distributed components. For each month, mean and standard deviations of the components were used to calculate the maximum chamber number and maximum test diameter. Based on these values, the natural chamber building rate (CBR) or diameter increase rate (DIR) could be estimated using the Michaelis-Menten function. CBR and DIR were inverted to estimate the ‘birthdate’ of all investigated individuals. Based on frequencies of these ‘birthdates’, main reproduction events could be detected and compared to the reproduction timing of other subtropical and tropical LBF taxa. Furthermore, peaks in reproduction could be linked to monsoon wet seasons (=“rainy seasons”) and winter rains.

## Introduction

The nummulitid foraminifer *Heterostegina depressa* belongs to the non-taxonomic (paraphyletic) group of larger benthic foraminifera (LBF), which are characterized by their symbiosis with phototrophic microalgae. Therefore, they are restricted to the photic zone of warm-temperate to tropic carbonate environments (Hallock, Röttger & Wetmore, 1991). Environmental constraints, like hydrodynamic energy, light penetration and nutrient influx, influence test morphology (Hohenegger, 2004; Briguglio & Hohenegger, 2009; Hohenegger, 2011). The complex test structures of LBFs have attracted scientific interest for a long time. A number of papers about their cell biology and ontogeny were published in the last decades (e.g., Beavington-Penney & Racey, 2004; Ferrandez-Canadell et al., 2014; Hallock, 1985; Hottinger, 1982; Lee et al., 1979). Moreover, information about ecological demands has been published in recent years; e.g., ecological niches and distribution (Hohenegger, 2004) in terms of water depth (Hottinger, 2006b; Renema, 2005), trophic resources (Hallock, 1988) and light intensity (Hohenegger, 2009). The main outcome reveals that light intensity is the most important factor controlling LBF’s depth distributions, where species occupy restricted niches along the light gradient (Hohenegger, 2000). For *H. depressa*, low light conditions are preferred (Nobes, Uthicke & Henderson, 2008), similar to other nummulitid species. However, it has been observed that *H. depressa* can adapt to strongly varying light conditions through test modification, (e.g., test flattening (Eder et al., 2016b)) or hiding in shadow regions to resist high light intensities. Further, Uthicke & Nobes (2008) showed that *H. depressa* does not show major changes in its distribution due to water quality. This explains the global presence in warm-temperate to tropic carbonate and in mixed-siliciclastic environments, from just below the water surface to about 100 m water depth (Hohenegger et al., 1999). Even though it occupies various niches in warm shallow-marine waters, *H. depressa* prefers to live in the study area around Sesoko-Jima (NW-Okinawa, Japan) semicryptically on reef structures and rubble in high energy regimes, or, similar to *Palaeonummulites venosus*, within the first few centimeters of sediment on sandy bottoms under lower energy regimes (Hohenegger, Yordanova & Hatta, 2000; Yordanova & Hohenegger, 2007).

Reproduction biology of *H. depressa* and especially its trimorphic life cycle has been studied in detail by Röttger, Krüger & De Rijk (1990). The succession of several schizontic, as well as the alternation of gamontic and agamontic generations has been observed in laboratory cultures (Krüger, 1994; Röttger, 1972a; Röttger, 1972b). The assumed morphological difference between megalospheric schizonts (A1) and gamonts (A2) based on laboratory cultures (Biekart et al., 1985; Leutenegger, 1977) has been recently documented in natural populations from Okinawa. Schizonts and gamonts of *H. depressa* exhibit a bathymetric separation due to hydrodynamics, which restrains gametes from forming zygotes in high energy environments (Eder, Hohenegger & Briguglio, 2017).

Growth of *H. depressa* has been thoroughly studied for initial growth stages (Röttger, 1974) and for later growth stages (Krüger, 1994; Röttger, 1972a). These studies are among the few that recorded growth in terms of diameter increase, as well as chamber number. The chamber number and maximal test diameter are the two commonly used characters to quantify growth in LBF. Test diameters are easily measurable using light microscopy, but chamber numbers becomes difficult due to the non-transparency of the central test part. In the last years, microCT has been frequently used for studying the morphology of naturally and laboratory-grown larger foraminifera to assess their growth (Speijer et al., 2008; Schmidt et al., 2013; Ferrandez-Canadell et al., 2014; Renema & Cotton, 2015; Briguglio et al., 2016).

Apart from quantification of growth (Krüger, 1994; Lietz, 1996; Röttger, 1972b), test diameter (TD) is an important character measured in paleontological studies to estimate water depth using thickness/diameter ratio (Cosovic, Drobne & Moro, 2004; Hallock, Forward & Hansen, 1986; Larsen & Drooger, 1977; Renema, 2005). Further, a detailed review on the thickness/diameter ratio and environmental parameters influencing it, as well as its importance in nummulitids has been made by Hohenegger (2004). The author’s hypothesis that the use of T/D ratios as bathymetric indicator is impeded in nummulitids since they don’t grow isometrically, contrary to *Amphistegina*, has been recently confirmed in studies on extant *H. depressa* (Eder, Hohenegger & Briguglio, 2018).

Based on the published laboratory investigations, mean chamber building rates (CBR) were estimated to study time-dependence (Hohenegger, Briguglio & Eder, 2014). These CBRs have been used to estimate growth oscillations in different nummulitid LBF, among those *H. depressa.* Cycles with periods hinting to tidal, lunar and meteorological forcing have been documented (Briguglio & Hohenegger, 2014; Eder, Briguglio & Hohenegger, 2016a). These results are possibly biased due to data obtained from laboratory cultures. Hence, to acquire unbiased information about growth oscillations, CBRs using individuals grown under natural conditions must be calculated. These topics and growth of LBF and foraminifera in general were further discussed in detail by Hohenegger (2018).

For studying population dynamics of larger benthic foraminifera, factors like the timing of reproduction, maximum life expectancy and growth rates are important to investigate the effects of seasonal and instantaneous environmental fluctuations on cell growth. As stated by Hohenegger, Briguglio & Eder (2014), population dynamics of LBF living in the eulittoral and uppermost sublittoral can be carried out easily (Fujita, Nishi & Saito, 2000; Hohenegger, 2006; Muller, 1974; Sakai & Nishihira, 1981; Zohary, Reiss & Hottinger, 1980). Investigations on species of the deeper sublittoral become more complex due to technical issues (e.g., sampling procedure) or extreme weather conditions (e.g., tropical cyclones). An additional issue can be the fixing of stable sampling stations needed to obtain comparable results within the investigation period. Hence, no field studies concerning asexual reproduction and longevity of mesophotic LBFs have been conducted so far. According to Wöger et al. (2016), only investigations with periods longer than 3 months are sufficient enough to gain information about life expectancy based on laboratory cultures. Among those few investigations, *H. depressa* with 12–13 months (Krüger, 1994; Röttger, 1972b), *Cycloclypeus carpenteri* with 12 months (Lietz, 1996) and *P. venosus* gamonts which had an average longevity around 17 months (Krüger, 1994) should be named.

For the study of reproduction timing, growth and life expectancy of LBF under natural conditions, the ‘natural laboratory’ (Hohenegger, Briguglio & Eder (2014) has been developed. This methodology has been already applied on some porcelaneous eulittoral species (e.g., *Peneroplis antillarum*, Hohenegger, 2006) and the sublittoral *P. venosus* (Kinoshita et al., 2017). Apart from that, a similar methodology has been applied on biostromes of the shallow-marine brachiopod *Magellania venosa* (Baumgarten et al., 2014).

In this study, the ‘natural laboratory’ approach will be applied on *H. depressa*. This approach has been introduced by Hohenegger, Briguglio & Eder (2014) to study growth of LBF in mesophotic environments where no regular observations are possible or are too expensive to conduct. This methodology uses population dynamic calculations to estimate growth of larger foraminifera. Monthly samples of a population of a LBF species are taken to estimate the current mean size (chamber-wise or in diameter) and to check for the presence of multiple generations for identifying peaks of main reproductions. By observing the mean size of population over one to one and half years in monthly intervals, a growth rate for test diameter and chamber number for the larger benthic foraminifera *H. depressa* has been modelled. The results are compared with known long-term cultures, especially Röttger (1972b), and Krüger (1994), as well as results of *P. venosus* (Kinoshita et al., 2017).

## Material and Methods

**Figure 1:**
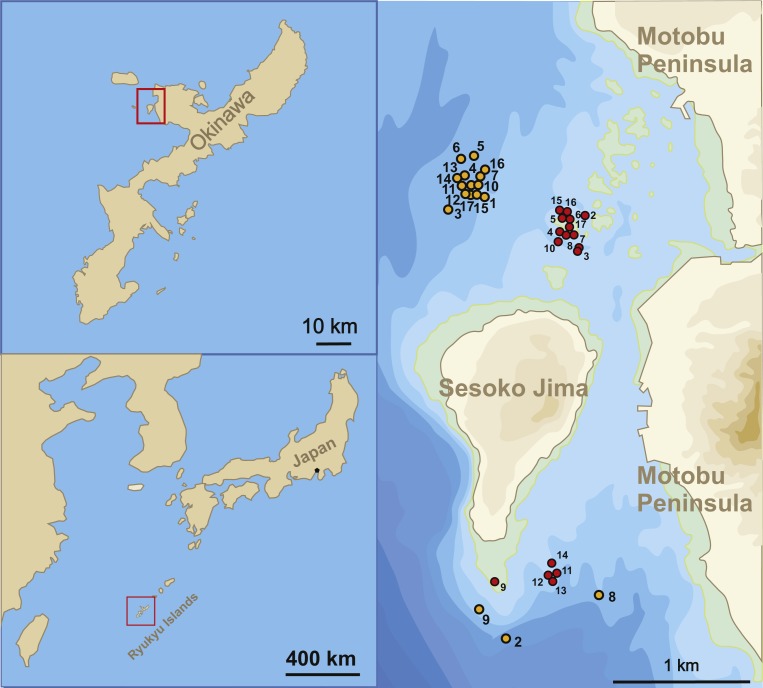
Sampling localities. Location of stations where samples were taken between April 23, 2014 and August 14, 2015.

The sample sites for this study were located in the Northwest and South of Sesoko-Jima, Okinawa, Japan (Fig. 1). The north-western sites were preferred during sampling due to the higher diversity of LBFs living on coarser substrate compared to the southern part with finer substrates (Hohenegger, 2004; Yordanova & Hohenegger, 2007). Because of bad weather conditions during winter time, samplings had to be relocated to the southern sampling area, where wind exposure is greatly reduced. Due to this protected location, sediment is finer (fine sand to silt) at 50 m (Table 1A). But LBF abundance was still sufficient for the presented study. Hence, *H. depressa* is generally more abundant in samples taken at the north-western site, where a higher proportion of rubble and coarse sand can be found (Hohenegger, 2004; Ujiié & Shioya, 1980).

**Table 1:** (A) Sampling date, coordinates, depth and on-site measurements for every sampling at ∼50 m water depth (temperature, salinity and pH), as well as main grain size, sample weight and megalospheric specimen number. (B) Sampling date, coordinates, depth and on-site measurements for every sampling ∼20 m water depth (temperature, salinity and pH), as well as megalospheric specimen. Main grain size and sample weight were not taken, since specimens were picked from reef rubble. Because *H. depressa* predominantly inhabits hard substrate, if available (Hohenegger et al., 1999).

A.										
Sample	Date	Longitude	Latitude	Depth	Temperature	Salinity	pH	Sediment		Number of individuals
								Main grain size	Weight in g	Gamonts / schizonts
1	23.04.2014	127°51.388′	26°40.086′	56	22,7	33,2		coarse sand	714,6	0
2	02.05.2014	127°52.243′	26°37.126′	46	22,3	28,6		fine sand / silt	381,8	5
3	09.05.2014	127°51.331′	26°40.039′	50	21,8	30,5	7,9	coarse sand	1183	49
4	30.05.2014	127°51.5160′	26°40.220′	54	23,3	31,9	7,9	coarse sand	216,2	11
5	18.07.2014	127°51.5324′	26°40.4240′	57,5	23,6	33,4	8,0	coarse sand	999	0
6	19.08.2014	127°51.4673′	26°40.4231′	56	26,2	32,2		coarse sand	349,5	18
7	10.09.2014	127°51.5281′	26°40.2410′	54	27,2	31,1		coarse sand	797,2	14
8	03.10.2014	127°52.2624′	26°37.4250′	41	26,9	30,1		fine sand / silt	1376,8	8
9	10.11.2014	127°51.4629′	26°37.3511′	41	24,7	30,4		coarse sand	1572,8	21
10	11.12.2014	127°51.517′	26°40.218′	47	23,5	30,8		coarse sand	515,1	16
11	16.01.2015	127°51.5101′	26°40.2142′	53,7	21,0	31,4		coarse sand	309,3	32
12	13.02.2015	127°51.5076′	26°40.1711′	57	20,1	31,7		coarse sand	488,4	12
13	04.03.2015	127°51.4727′	26°40.2670′	57	22,0	30,7		coarse sand	1055,4	6
14	15.04.2015	127°51.4540′	26°40.2362′	58	23,5	30,8	8,3	coarse sand	505,6	32
15	18.05.2015	127°51.5099′	26°40.2756′	55	22,9	31,3	8,0	coarse sand	267,1	11
16	11.06.2015	127°51.6201′	26°40.3148′	56,5	24,0	30,6		coarse sand	573,5	16
17	14.07.2015	127°51.5144′	26°40.1600′	50	27,4	29,9		coarse sand	229,1	42

B.								
Sample	Date	Longitude	Latitude	Depth	Temperature	Salinity	pH	Number of individuals
								Gamonts / Schizonts
1	02.05.2014	26°37.2000′	127°51.6350′	21	22,3	35,2		41
2	09.05.2014	26°39.7060′	127°52.2930′	25	21,9	35,1		36
3	30.05.2014	26°39.9089′	127°52.1564′	21	23,0	35,2	7,9	21
4	18.07.2014	26°39.9362′	127°52.1641′	25	26,0	31,1	8,1	21
5	19.08.2014	26°39.9351′	127°52.1659′	26	28,0	30,9	7,9	41
6	10.09.2014	26°39.9091′	127°52.1580′	27	28,2	30,4	8,1	37
7	20.10.2014	26°39.9080′	127°52.1612′	23,5	25,6	30,3	8,1	55
8	10.11.2014	26°37.4079′	127°51.5399′	22,7	24,8	30,5		11
9	11.12.2014	26°39.9008′	127°52.1523′	21,5	23,3	30,7		36
10	16.01.2014	26°37.4598′	127°51.8458′	22	20,9	31,4		7
11	13.02.2015	26°37.4445	127°51.8420	21,7	20,1	31,4		15
12	04.03.2015	26°37.4597′	127°51.8360′	23	21,8	30,6		52
13	15.04.2015	26°37.4950′	127°51.8422′	21	23,3	30,7		16
14	18.05.2015	26°39.9471′	127°52.1600′	27	23,2	31,1		15
15	11.06.2015	26°39.9430′	127°52.1642′	25,3	25,5	30,3		47
16	14.07.2015	26°39.9160′	127°52.1652′	21	27,8	29,5		52

Samples, including environmental parameters (temperature and salinity) were taken between April 23, 2014 and July 17, 2015 in seventeen consecutive monthly samplings following the methods of Hohenegger, Briguglio & Eder (2014). Sampling were carried out at ∼20 and ∼50 m water depth (Table 1). Since *H. depressa* exhibits a broad depth distribution in the investigated area, populations of both depths have been used to apply the ‘natural laboratory’ approach. The initially exercised monthly sampling intervals couldn’t be maintained throughout the year because of unfavourable weather conditions (e.g., north-westerly winter winds, tropical cyclones), resulting in uneven (∼1–2 weeks around the preferred sampling date) sampling intervals.

Four samples at each sampling depth (20 and 50 m) were scooped from the uppermost centimetres of sediment using plastic boxes. Fine sediment (silt and mud) was decanted from 50 m samples and the coarser fractions containing the living LBF were moved into shallow boxes. Samples from 20 m mainly contained coral rubble. Here, easily visible specimens were picked using feather steel forceps. The remaining foraminifera were brushed from the rubble into shallow boxes using a soft brush. All samples rested for a period of 24 h, after which living specimens are easily recognizable by their coloured protoplasm due to the symbionts spread into the final chambers. A small part of the picked living specimens were selected for growth investigations under laboratory conditions. The tests of the remaining population, as well as sediment samples, were washed with fresh water and dried. For a more detailed description of sampling and sample processing refer to Wöger et al. (2016).

All specimens of *H. depressa* used in this analysis were scanned using the micro-CT facility at the University of Vienna (Skyscan 1173 at 100 kV, 80 µa, Aluminium-Filer with average pixel size of 8 µm) and investigated chamber number (NoC) and test diameter (TD) were measured, which enabled the calculation of time-related growth based on population dynamics. Primary data can be accessed via following link; https://doi.org/10.5281/zenodo.1477635.

### Analysis

In this study, only megalopsheres (gamonts and schizonts) have been investigated because microspheres didn’t occur frequently enough to analyse their growth by frequency distributions. To infer the chamber building rate (CBR) and test diameter increase rate (DIR) from the sampled populations, the ‘natural laboratory’ approach (Hohenegger, Briguglio & Eder, 2014) has been used.

The chamber number (NoC) is counted including nepiont (proloculus and deuteroloculus), while the maximal test diameter (TD) is measured through the centre of the proloculus. NoCs are processed as natural numbers, while TDs are transformed using the natural logarithm due to the nonlinear (logarithmic) test growth.

Chamber number and test diameter of the seventeen samples were illustrated as frequency diagrams using identical intervals along the abscissa. The illustration using densities (frequency per sediment weight) for 50 m samples as done in Hohenegger, Briguglio & Eder (2014) proved to be difficult, due to the different sampling areas (NW and S) with different sedimentary composition at the same water depths.

Since distribution parameters mean ($\bar {x}$) and standard deviation (sd), which are important to calculate growth NoC and TD, are constant when using frequencies, densities or proportions, absolute frequencies can be used without becoming biased.

Initially, the frequency distribution of each sample has been checked for normality by Chi-square goodness-of-fit test. If samples significantly deviate from normal distributions, they have been decomposed into normally distributed components using nonlinear regression based on numerical mathematics (IBM SPSS 22).

The maximum NoC or TD at time *t* is calculated based on the mean and standard deviation of component *j* using

(1) \begin{eqnarray*}{m}_{jt}=\bar {{x}_{jt}}+3{s}_{jt}^{\ast }.\end{eqnarray*}

The normalized standard deviation s* is based on the mean coefficient of variance (*CV*) and calculated in accordance to Hohenegger, Briguglio & Eder (2014) by

(2) \begin{eqnarray*}{s}_{jt}^{\ast }=C{V}_{mean}/\bar {{x}_{jt}}.\end{eqnarray*}

By illustrating the components as a function of time within the time interval of 15 months (May 2014 to July 2015), four megalospheric generations, maximum two per year, were identified. These two generations increase continuously through the investigation period with the same growth trend but with different onsets. The onset of one generation is the temporal interval before the date of the components with the lowest estimated maximum *m*_*j*_. This onset is characterized by chamber numbers of *m*_*j*1_ = 2 and *m*_*j*2_ = 3 as observed by Röttger (1974) in cultures of *H. depressa*. To estimate initial values for test diameter, the mean value over all investigated individuals at chamber number = 2 and chamber number = 3 were measured and resulted in *m*_*j*1_ = 293.7 µm and *m*_*j*2_ = 346.5 µm for 20 meters’ populations and *m*_*j*1_ = 296.4 µm and *m*_*j*2_ = 347.6 µm for 50 meters’ population.

Subsequent, the CBR was estimated using Eq. (3) for both generations within one year.

(3) \begin{eqnarray*}{m}_{jt}={m}_{\mathrm{jmax}}\ast t/ \left( {b}_{j}+{t}_{j} \right) \end{eqnarray*}

where *m*_jmax_ represents the growth asymptote (e.g., maximal possible chamber number or test diameter) and *b*_*j*_ the *t*-value where *m*_jmax_∕2 is reached. Eq. (3) is similar to the Michaelis–Menten function (Michaelis & Menten, 1913), which intersects the origin. Due to the maturo-evolute growth of *H. depressa* (Banner & Hodgkinson, 1991) which exhibits, contrary to Eq. (3), a rather slow initial growth and very slow reduction in later growth stages, a generalized logistic Michaelis–Menten function (Lopez et al., 2000) has to be used for estimating DIR

(4) \begin{eqnarray*}{m}_{jt}= \left( {m}_{j0}\ast {b}_{j}^{{c}_{j}}+{m}_{\mathrm{jmax}}\ast {t}^{{c}_{j}} \right) /({b}_{j}^{{c}_{j}}+{t}^{{c}_{j}})\end{eqnarray*}

where *b*_*j*_ and *c*_*j*_ are function constants, *m*_*j*0_ the nepiontic diameter and *m*_jmax_ the maximal diameter. The duration of the onset for the CBR has been estimated using an iterative process, where the onset has been initially fitted with 10 days and increased up to 70 days in 5-day steps. Adjacent, the fit of the estimated function calculated using Eq. (5) to the observed *m*_*j*_-values was tested by a reduced Chi-square goodness-of-fit test. The interval with the best Chi-square scores has been again tested with day-wise steps to find the exact length of onset. The onset with the best fit to the experimental data has been used to estimate the parameters of the Michaelis–Menten function *m*_jmax_ and *b*_*j*,_which are used in the succeeding analysis. The same length of onset time has been applied for the DIR.

Parameters *m*_*j*__max_ and *b*_*j*_ of the first generation, which exhibits the higher *m*_jmax_ values have been used to estimate the birthdate *t*_0_ of each specimen *i*. NoC of each specimens *i* at sampling date *t*_*j*_ defines the birthdate estimated by Eqs. (5) (NoC) or (6) (TD).

(5) \begin{eqnarray*}t \left( 0 \right) =t \left( i \right) -No{C}_{i}\ast B/ \left( {m}_{\mathrm{jmax}}-No{C}_{i} \right) \end{eqnarray*}

(6) \begin{eqnarray*}t \left( 0 \right) =t \left( i \right) -((T{D}_{i}-{m}_{j0})\ast {b}_{{j}^{{c}_{j}}}/{m}_{\mathrm{jmax}}-T{D}_{i})^{ \frac{1}{{c}_{j}} }.\end{eqnarray*}

Birthdates of all analyzed specimens are illustrated as frequency diagrams with monthly intervals. For samples from 50 m water depth simple counts of densities (count = 1) are biased by differing sample size of. Hence, a transformation of counts of densities (count*) per specimen *i* of sample *k* was used (Eq. (7)).

(7) \begin{eqnarray*}{\mathrm{count}}_{ik}^{\ast }=1/\mathrm{sample}~{\mathrm{size}}_{k}\end{eqnarray*}

No volume measurements of reef rubble were acquired for samples from 20 m, therefore simple counts had to be used for frequency distributions.

Lomb periodograms (Press et al., 1992) were used to scan for significant periods within the frequencies of birthdates and compared with sinusoidal regression models based on Nyquist frequencies ( Shannon, 1949) and harmonic series (Hammer, Harper & Ryan, 2001).

The estimation of longevity have not been calculated in accordance to Hohenegger, Briguglio & Eder (2014), but rather by computing the maximum difference in days between individual reproduction date and sampling date (Kinoshita et al., 2017)

(8) \begin{eqnarray*}\mathrm{max} \left[ t \left( i \right) -{t}_{i} \left( 0 \right) \right] .\end{eqnarray*}

More complex statistical investigations (e.g., numerical mathematical decomposition and/or fitting of the Michaelis–Menten functions) have been done using IBM SPSS Statistics 22 and Past 3.02 (Hammer, Harper & Ryan, 2001), while the remaining calculations were performed in Excel Microsoft Office 2013.

## Results

The 422 megalospheric specimens of *Heterostegina depressa* from ∼20 m water depth, which should be referred as schizonts according to Eder, Hohenegger & Briguglio (2017), show almost no significant correlation between NoC and TD (Table 2). Exemptions are samples with high numbers of small or large specimens (e.g.; August 19, 2014, September 10, 2014, December 11, 2014, July 14, 2015), where significant correlation could be observed. All investigated samples exhibit relatively low *R^2^* values. Further, frequency distributions of all eleven samples used for the natural laboratory approach show statistically significant deviation from normal distribution based on the Chi-square scores (see Table 2).

On the contrary, the 377 megalospheric specimens sampled at ∼50 m water depth, which mostly consist of gamonts, show highly significant correlation between NoC and TD with relatively high *R^2^* scores except in one sample (May 9, 2014). Similar to samples from 20 m, all twelve investigated samples show significant deviation from normal distribution (see Table 3).

**Table 2:** Correlation between measured parameters and test for normal-disribution (20 m). Correlation between chamber number and the logarithm of the test diameter at the sampling sites used for the ‘natural laboratory’ at 20 m tested for significance (high correlation is present in sample 19.08.2014, 10.09.2014, 11.12.2014, 14.07.2014) and testing normal distribution of chamber number and the logarithm of test diameter by chi-square tests.

20 m							
Date	*n*	Correlation		Chamber number		Test diameter	
		*R*^2^	*p*(H0)	*x*^2^	*p*(H0)	*x*^2^	*p*(H0)
02.05.2014	41	0,06	0,111	39,90	3,30E–06	1318,73	1,38E–278
30.05.2014	25	0,02	0,524	57,20	2,04E–09	13,90	0,036
18.07.2014	21	0,00	0,822	21,53	0,004	33,85	3,82E–05
19.08.2014	41	0,45	1,43E–06	147,03	1,73E–27	26,86	0,001
10.09.2014	37	0,14	0,021	23,23	0,002	11,89	0,058
20.10.2014	55	0,05	0,117	19,38	0,008	21,42	0,004
11.12.2014	36	0,23	0,003	18,89	0,009	23,16	0,002
13.02.2015	15	0,19	0,100	132,54	1,69E–24	20,10	0,006
03.03.2015	52	0,00	0,860	16,66	0,017	48,48	8,94E–08
11.06.2015	47	0,08	0,054	48,77	7,91E–08	11,82	0,058
14.07.2014	52	0,74	2,59E–16	25,00	0,001	16,72	0,017
		F	p(same slope)				
		13,39	2,62E–20				

**Table 3:** Correlation between measured parameters and test for normal-disribution (50 m). Correlation between chamber number and the logarithm of the test diameter at the used for the ‘natural laboratory’ at 50% m sampling sites tested for significance (high correlation is present in sample 9.05.2015) and testing normal distribution of chamber number and the logarithm of test diameter by chi-square tests.

50 m							
Date	*n*	Correlation		Chamber number		Test diameter	
		*R*^2^	*p*(H0)	*x*^2^	*p*(H0)	*x*^2^	*p*(H0)
09.05.2014	49	0,13	0,019	125,87	3,96E–23	27,68	4,13E–04
30.05.2014	11	0,73	0,008	51,64	2,30E–08	33,01	5,34E–05
19.08.2014	18	0,83	1,63E–07	26,74	3,10E–04	243,38	1,21E–47
10.09.2014	14	0,62	0,001	52,15	1,85E–08	30,26	1,55E–04
10.11.2014	21	0,68	3,72E–06	50,00	4,66E–08	287,13	6,82E–57
11.12.2014	16	0,62	3,08E–04	41,18	1,94E–06	287,13	6,82E–57
16.01.2015	32	0,88	1,57E–15	28,37	3,19E–04	35,80	1,76E–05
13.02.2015	12	0,77	1,60E–04	29,24	2,30E–04	38,54	5,77E–06
15.04.2015	32	0,68	8,15E–09	49,74	5,21E–08	48,83	7,71E–08
18.05.2015	11	0,61	0,004	30,19	1,60E–04	5,20	0,091
11.06.2015	16	0,82	1,18E–06	43,34	7,89E–07	40,12	3,02E–06
14.07.2014	42	0,83	4,39E–17	23,66	0,002	24,02	0,002
		F	p(same slope)				
		2,2	0,014				

Samples with a specimen number less than 10 have not been included in the ‘natural laboratory’.

**Figure 2:**
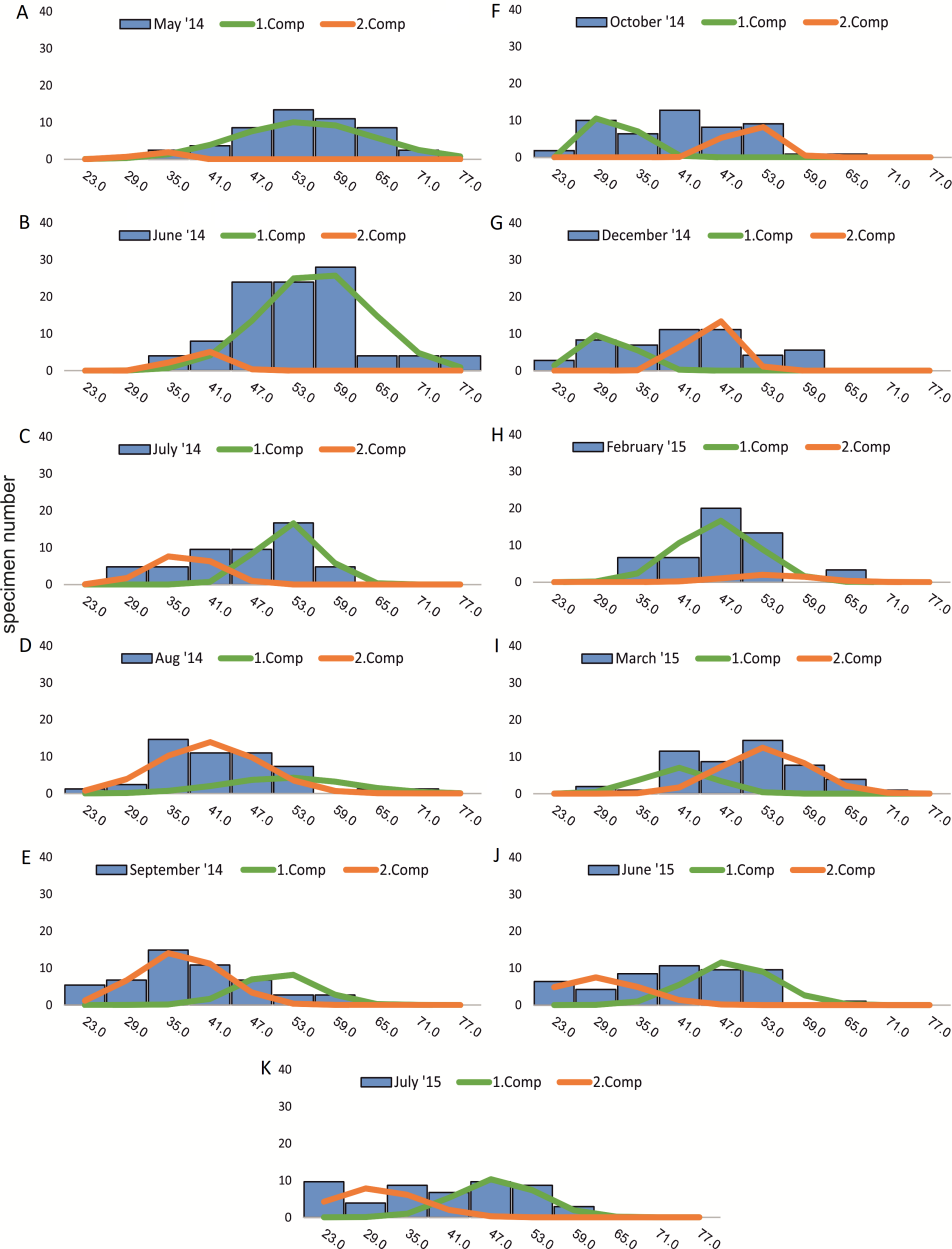
Decomposition of frequency distributions chamber number (20 m). Decomposition of frequency distributions into normal-distributed components based on chamber number at 20 m water depth. Histograms are standardized to 50 specimens. (A) Sample May 2014; (B) Sample June 2014; (C) Sample July 2014; (D) Sample August 2014; (E) Sample September 2014; (F) Sample October 2014; (G) Sample December 2014; (H) Sample February 2015; (I) Sample March 2015; (J) Sample June 2015; (K) Sample July 2015.

**Figure 3:**
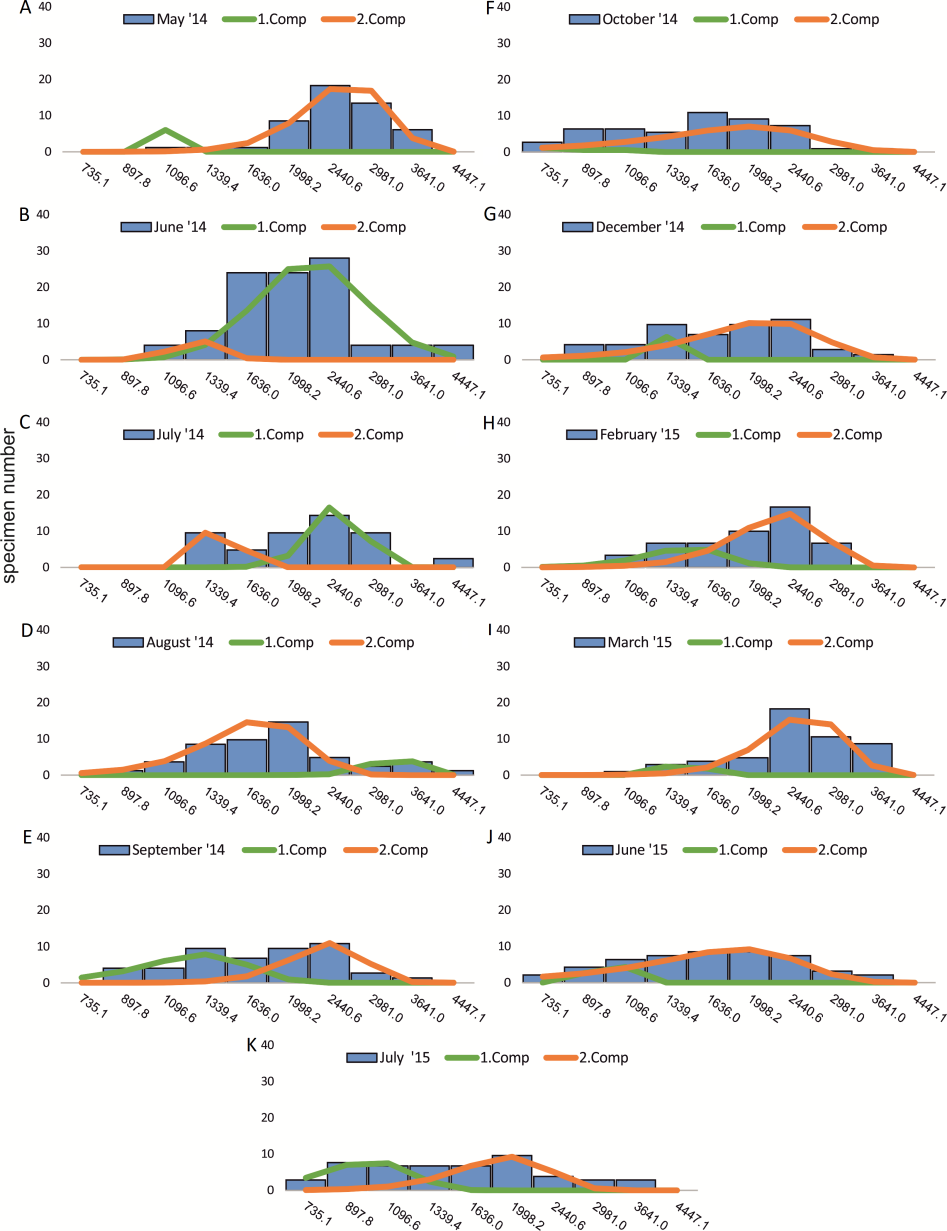
Decomposition of frequency distributions test diameter (20 m). Decomposition of frequency distributions into normal-distributed components based on test diameter at 20 m water depth. Histograms are standardized to 50 specimens. (A) Sample May 2014; (B) Sample June 2014; (C) Sample July 2014; (D) Sample August 2014; (E) Sample September 2014; (F) Sample October 2014; (G) Sample December 2014; (H) Sample February 2015; (I) Sample March 2015; (J) Sample June 2015; (K) Sample July 2015.

**Figure 4:**
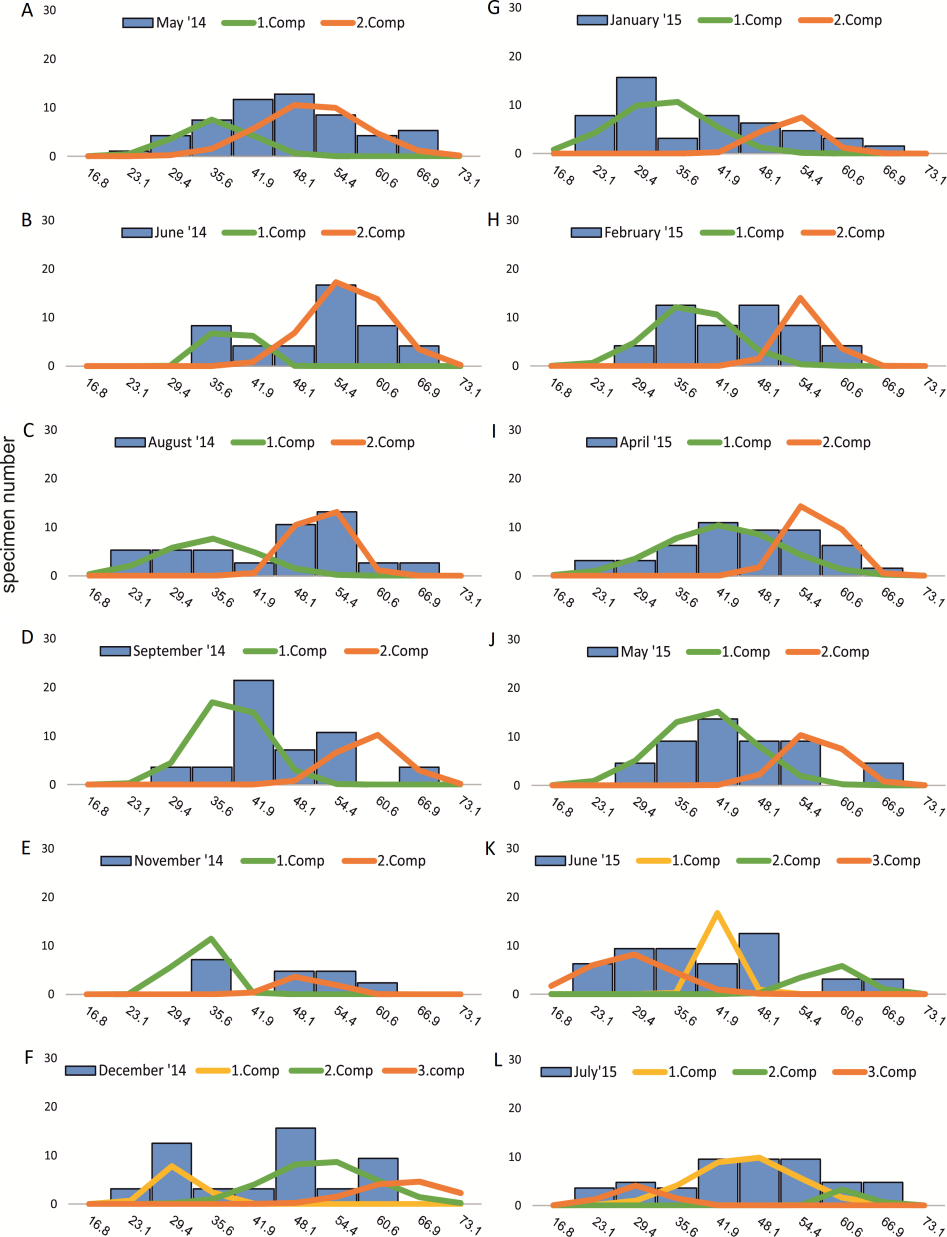
Decomposition of frequency distributions chamber number (50 m). Decomposition of frequency distributions into normal-distributed components based on chamber number at 20 m water depth. Histograms are standardized to 50 specimens. (A) Sample May 2014; (B) Sample June 2014; (C) Sample August 2014; (D) Sample September 2014; (E) Sample November 2014; (F) Sample December 2014; (G) Sample January 2015; (H) Sample February 2015; (I) Sample April 2015; (J) Sample May 2015; (K) Sample June 2015; (L) Sample July 2015.

**Figure 5:**
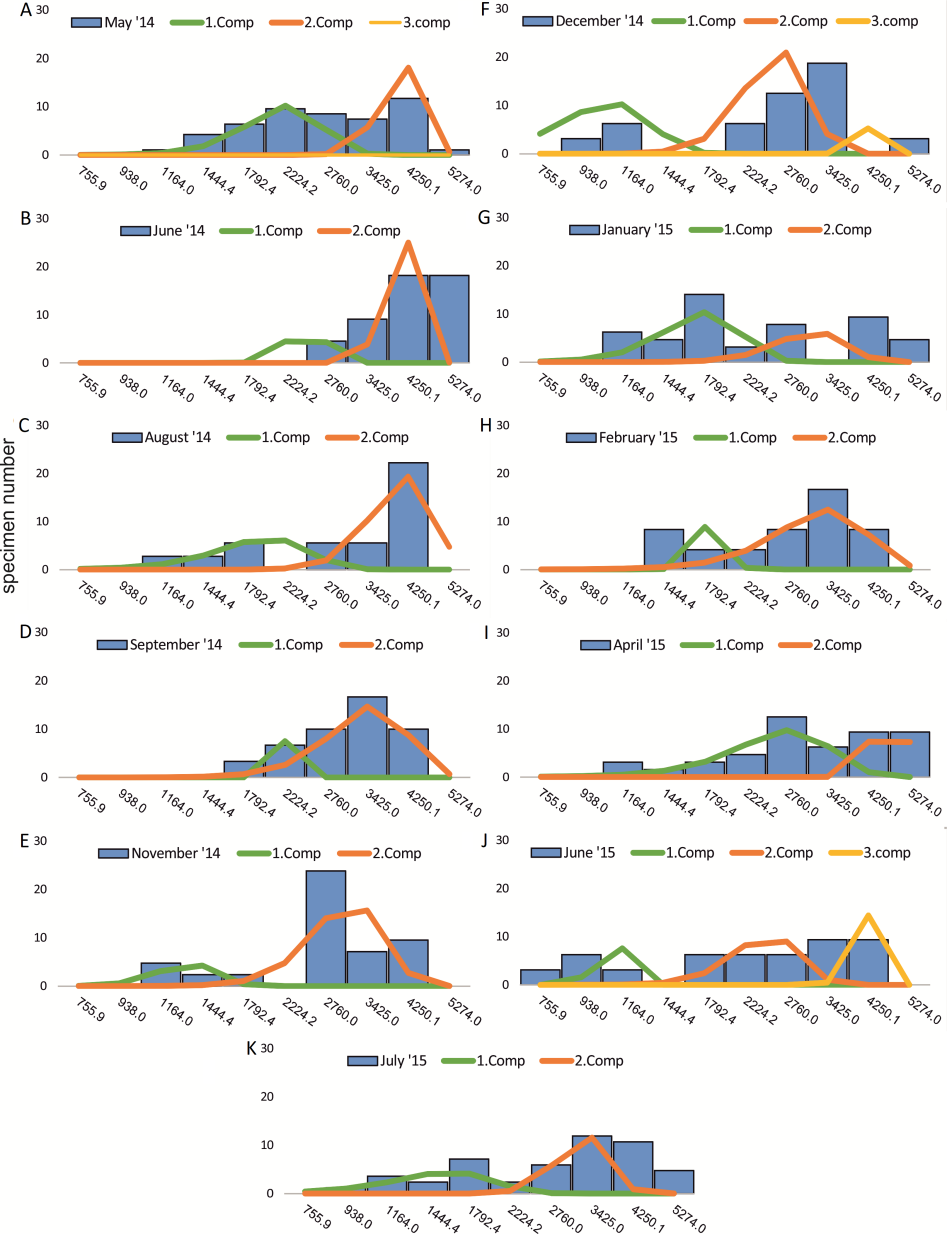
Decomposition of frequency distributions chamber number (50 m). Decomposition of frequency distributions into normal-distributed components based on test diameter at 20 m water depth. Histograms are standardized to 50 specimens. (A) Sample May 2014; (B) Sample June 2014; (C) Sample August 2014; (D) Sample September 2014; (E) Sample November 2014; (F) Sample December 2014; (G) Sample January 2015; (H) Sample February 2015; (I) Sample April 2015; (J) Sample May 2015; (K) Sample June 2015; (L) Sample July 2015.

**Figure 6:**
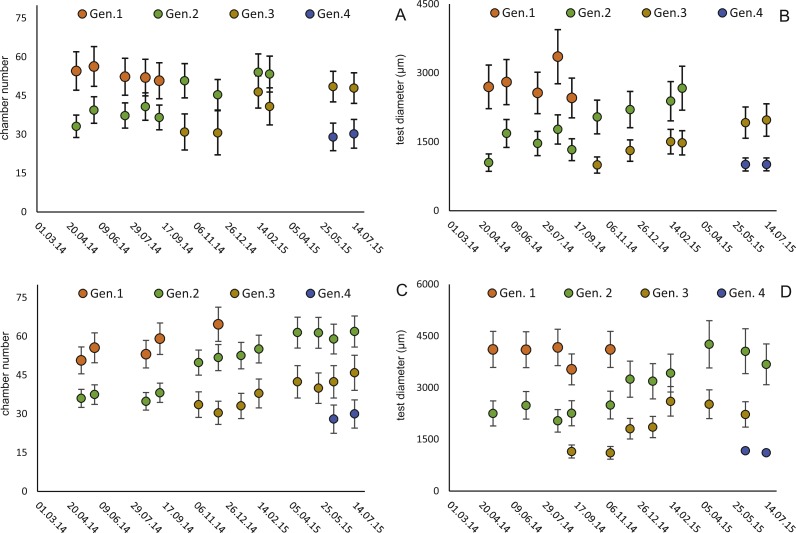
Illustration of mean and standard deviation of the monthly components per generation. 20 m samples; Generation 2 (A) and Generation 3 (B) are more or less completely represented, similar at 50 meter’s samples’ Generation 2 (C). Only Generation 3 (D) is slightly truncated.

Within the frequency distributions of the investigated months up to three components can be differentiated (Figs. 2 and  3 for 20 meter samples and Figs. 4 and 5 for 50 m samples), The decomposition could be computed on all monthly samples given in Tables 2 and 3. Parameters of the normally distributed components $\bar {{x}_{j}}$(mean), *s*_*j*_ (standard deviation) and *d* (density) are given in Tables 4 (20 m) and 5 (50 m) and illustrated in Fig. 6 for both water depth stations.

Four different generations of megalospheric *H. depressa* could be observed over the complete sampling period. Generation 2 and Generation 3 cover most of the investigation period. Generations 1 and 4 rather record the very late and early stages of growth. Generation 1 at 20 m is observed from the start of the investigation period up to August 2014 with the largest specimens (in regards to NoC and TD), while generation 4 is first observed in June 2015 with rather small specimens. A quite similar pattern can be observed for generations from 50 m water depth. However, Generation 1 is present later in the year (up to December) but is missing in the November sample.

**Figure 7:**
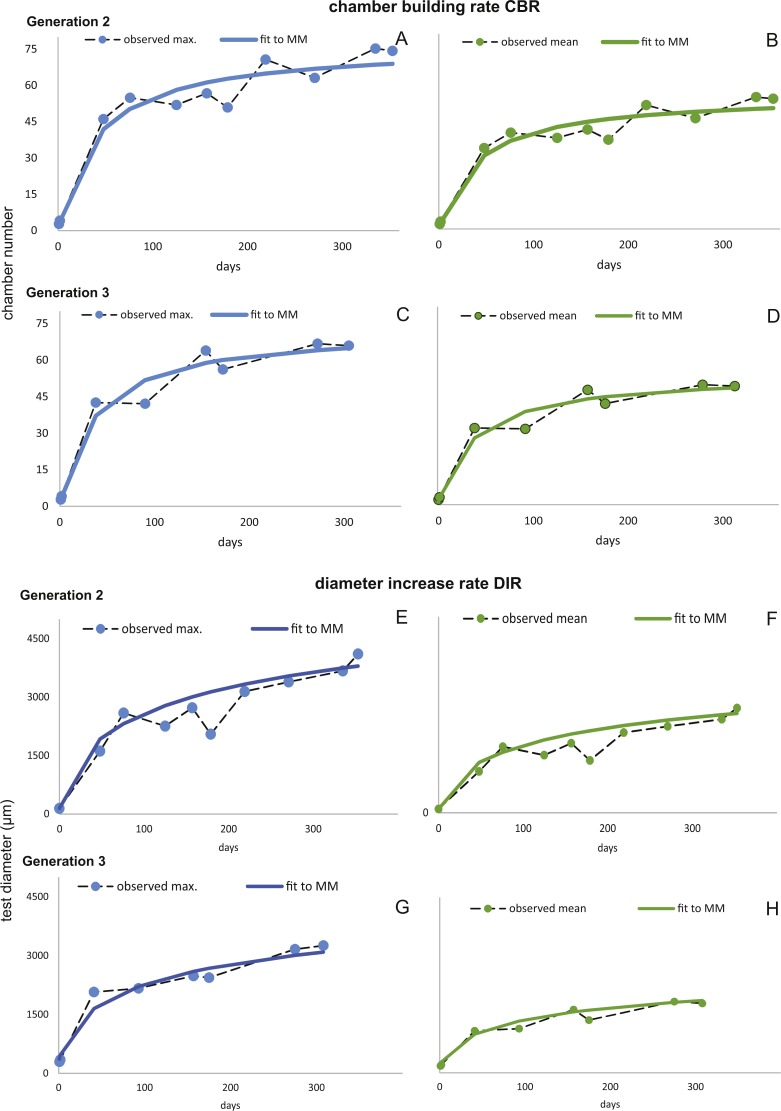
Fit of CBR and DIR in both generations to mean and maximal values by Michaelis-Menten or generalized MM functions at 20 m. CBR; Generation 2 maximum (A), Generation 2 mean (B), Generation 3 maximum (C), Generation 3 mean (D). DIR; Generation 2 maximum (E), Generation 2 mean (F), Generation 3 maximum (G), Generation 3 mean (H).

**Figure 8:**
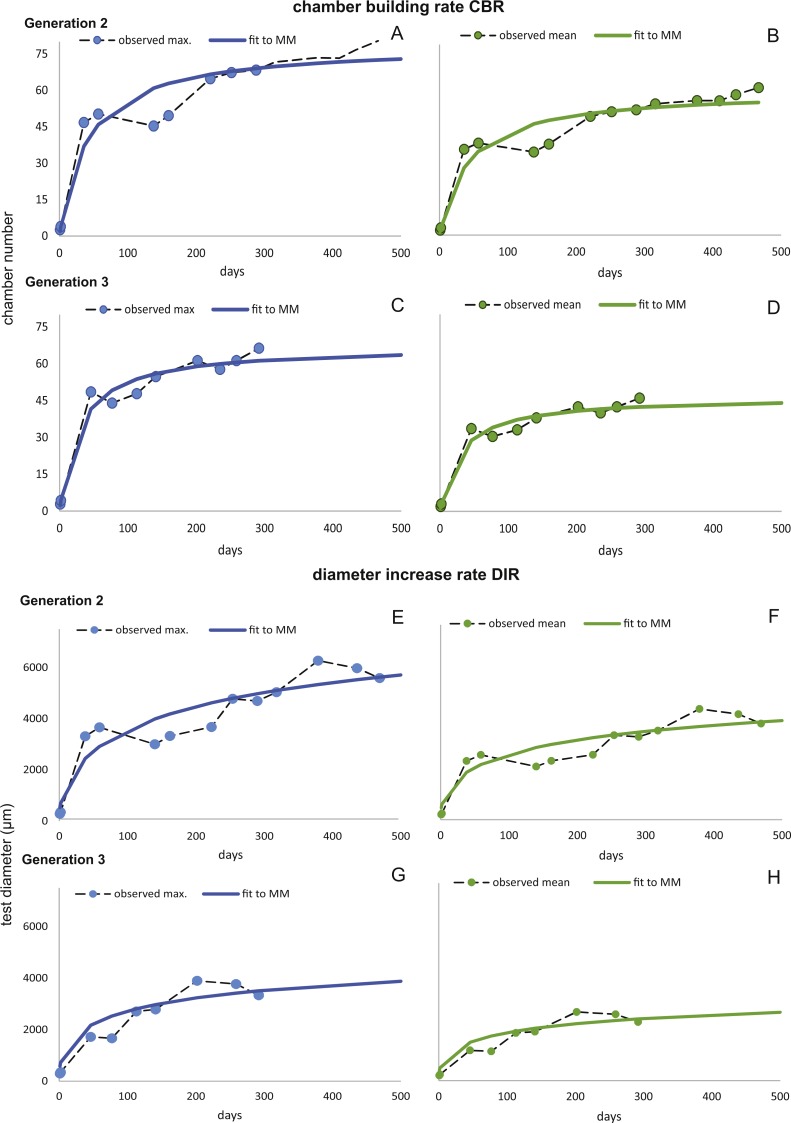
Fit of CBR and DIR in both generations to mean and maximal values by Michaelis-Menten or generalized MM functions at 50 m. CBR; Generation 2 maximum (A), Generation 2 mean (B), Generation 3 maximum (C), Generation 3 mean (D). DIR; Generation 2 maximum (E), Generation 2 mean (F), Generation 3 maximum (G), Generation 3 mean (H).

Fitting Eq. (3) (for NoC) and Eq. (4) (for TD) using the transformed maximal values m_*jt*_ (Eq. (1)) results in significant fits for both characters. The estimated chamber building rates and diameter increase rates as well as the corresponding statistics for Generations 2 and 3 for the population for 20 m and 50 m population are given in Figs. 7 and 8. Function parameters of CBR and DIR show no significant differences between Generations 2 and 3 at both water depths (see the corresponding Student’s test in the Supplemental Information 1).

**Table 4:** Decomposed normal-distributed components (20 m). Component parameters density, mean and standard deviation related to the sampling time during the investigation period for NoC and TD at 20 m.

	Component 1			Component 2		
	Density	Mean	s.d.	Density	Mean	s.d.
** Chamber number (NoC)**						
02.05.2014	8	54.6	9.79	2	33.2	2.54
30.05.2014	7	56.3	7.85	1	39.5	3.25
18.07.2014	7	52.4	4.53	4	37.3	4.64
19.08.2014	4	52.0	8.91	11	40.8	7.38
10.09.2014	7	50.8	5.35	11	36.6	6.07
20.10.2014	13	31.0	3.86	11	50.8	3.32
11.12.2014	8	30.6	3.82	11	45.4	3.35
13.02.2015	5	46.5	5.79	1	54.1	6.02
03.03.2015	7	40.9	5.09	13	53.4	6.17
11.06.2015	11	48.5	6.05	7	29.1	6.46
14.07.2015	11	48.0	5.98	8	30.3	6.46
** Test diameter (TD)**						
02.05.2014	9	1049.7	43.14	16	2697.8	519.57
30.05.2014	6	1683.8	52.77	5	2801.8	782.35
18.07.2014	10	1466.9	91.93	8	2567.0	306.56
19.08.2014	13	1772.3	403.07	4	3354.0	374.11
10.09.2014	6	1330.9	322.40	8	2456.5	429.63
20.10.2014	3	997.2	54.20	8	2043.2	693.81
11.12.2014	5	1312.4	67.16	8	2203.2	612.81
13.02.2015	2	1504.2	282.67	4	2385.7	487.16
03.03.2015	8	1479.8	91.28	18	2669.2	503.46
11.06.2015	8	1009.7	71.64	9	1919.5	638.80
14.07.2015	8	1010.9	210.80	10	1976.7	424.92

**Table 5:** Decomposed normal-distributed components (50 m). Component parameters density, mean and standard deviation related to the sampling time during the investigation period for NoC and TD at 50 m.

	Component 1			Component 2			Component 3		
	Density	Mean	s.d.	Density	Mean	s.d.	Density	Mean	s.d.
**Chamber number (NoC)**									
09.05.2014	14	36.0	5.46	7	50.7	9.59			
30.05.2014	3	38.6	2.87	4	56.3	5.80			
19.08.2014	3	29.2	7.26	6	51.8	3.82			
10.09.2014	5	38.2	5.14	3	59.1	4.81			
10.11.2014	6	33.6	3.04	2	49.9	3.68			
11.12.2014	3	30.4	3.28	3	51.8	7.80	5	64.7	6.84
16.01.2015	7	33.1	7.13	5	52.6	4.10			
13.02.2015	3	38.0	6.06	3	55.1	3.29			
15.04.2015	7	42.5	8.92	11	56.5	1.95			
18.05.2015	3	40.0	7.03	11	56.5	3.95			
11.06.2015	6	42.5	2.36	3	56.4	4.57	8	28.4	6.49
14.07.2015	9	46.0	7.66	3	61.9	2.70	4	29.6	4.19
**Test diameter (TD)**									
09.05.2014	14	36.0	5.46	7	50.7	9.59	
30.05.2014	3	38.6	2.87	4	56.3	5.80			
19.08.2014	3	29.2	7.26	6	51.8	3.82			
10.09.2014	5	38.2	5.14	3	59.1	4.81			
10.11.2014	6	33.6	3.04	2	49.9	3.68	5	64.7	6.84
11.12.2014	3	30.4	3.28	3	51.8	7.80	
16.01.2015	7	33.1	7.13	5	52.6	4.10			
13.02.2015	3	38.0	6.06	3	55.1	3.29			
15.04.2015	7	42.5	8.92	11	61.5	1.95			
18.05.2015	3	40.0	7.03	3	61.4	3.57			
11.06.2015	6	42.5	2.36	2	59.0	4.21	8	28.4	6.49
14.07.2015	9	46.0	7.66	3	61.9	4.70	4	29.6	4.19

**Figure 9:**
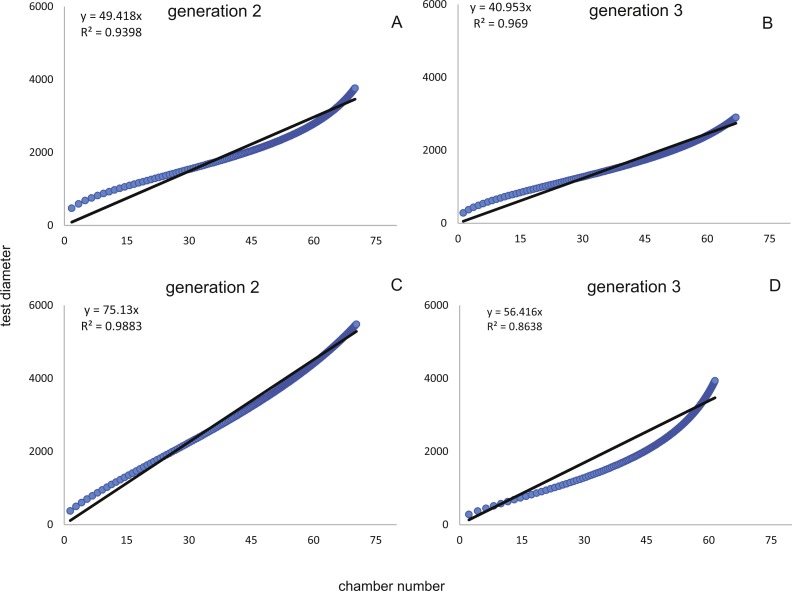
Correlation between CBRs (*x*-axis) and DIRs (*y*-axis). For Generation 2 (A) and 3 (B) at 20 m and for Generation 2 (C) and 3 (D) at 50 m.

In comparison CBR and DIR are highly correlative, yet don’t exhibit a linear correlation. Deviations from a linear correlation can be especially well observed in the initial and later test parts. This pattern is stronger in specimens from 20 m water depth than those from the deeper samples, as seen in Fig. 9.

For further analyses, the parameters of CBRs and DIRs of Generation 2 have been used for samples from both water depths.

### Inference of reproduction time

**Figure 10:**
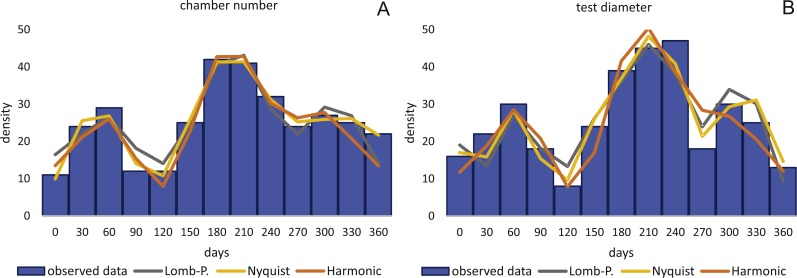
Illustration of histograms for estimated birthdates of all investigated specimens. By inversion of the CBR (A) and DIR (B) at 20 m. Density is given as simple counts.

**Figure 11:**
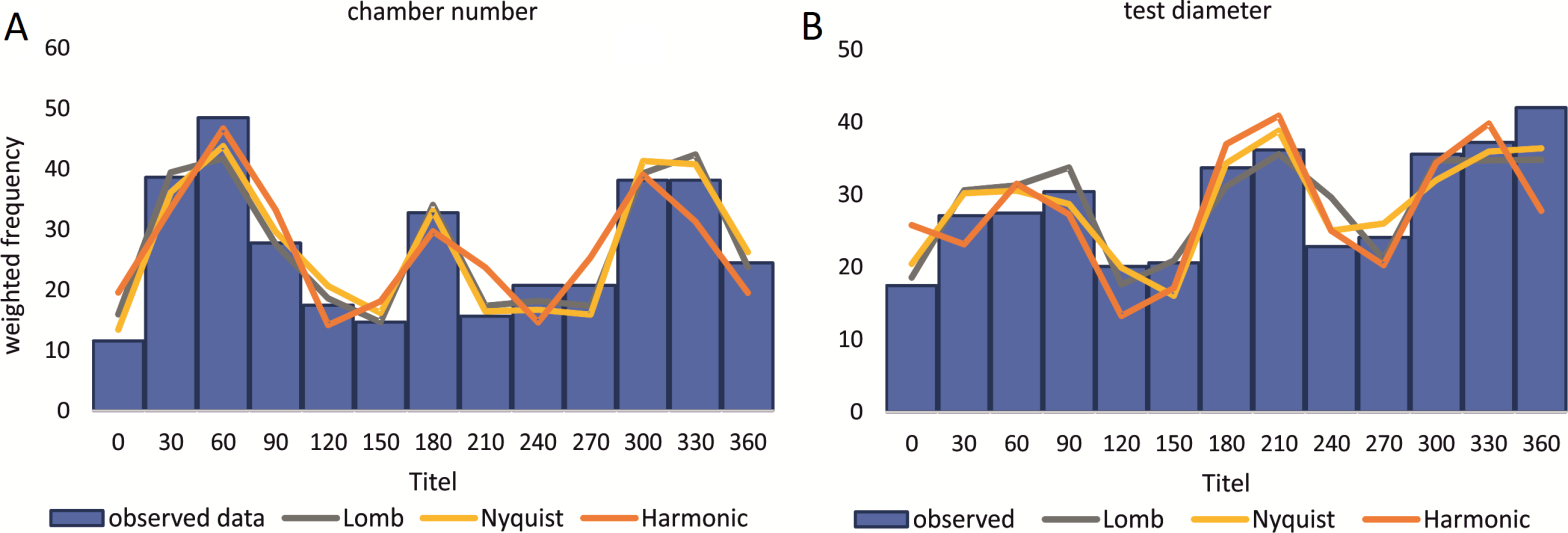
Illustration of histograms for estimated birthdates of all investigated specimens. By inversion of the CBR (A) and DIR (B) at 50 m. Density is weighted by sediment weight.

Birthdates for every sampled specimen were inferred based on a) the chamber building rate and b) on the diameter increase rate. Estimated birthdates of the 20 meters’ population are given as histograms with monthly intervals using simple counts (Figs. 10A, 10B) to illustrate peaks of reproduction and test for periodicities in reproduction. For the 50 meters’ population, histograms are given as counts normalized by sediment weight (Eq. (7); Figs. 11A, 11B).

CBR’s histograms for 20 m clearly illustrate two major reproduction phases over the year, one in summer (July–August) and one in winter (February–March and November–December) with the summer reproduction being dominant.

At 50 m reproduction timing based on the CBR does not change between histograms using simple or standardized counts. Reproduction peaks occur around similar times, in summer (July–August) and winter (February–March and November–December). Reproduction events are more or less equally expressed, with slightly increased winter peaks.

Sinusoidal regression analysis on CBR’s and DIR’s histograms is acquired by the sum of sinusoids using the most significant periods of the Lomb periodogram, the Nyquist frequencies (ESM3) and the harmonic series. The best fit is gained using Nyquist frequencies and the Lomb periodogram, while harmonic series shows the worst fit. The sum-of-sinusoids is not identically repetitive over several years for Lomb periodogram and Nyquist frequencies and similar patterns continue if the cycles have convergent phases. Continuity over several years is only given by the harmonic series, hence they should be used if long-term trends are to be predicted.

Amplitudes of the sinusoidal functions based on CBR depicts the importance of oscillations. The population from 20 m exhibit, according to the Lomb periodogram, periods at 261.8 days, 137.1 days and 80 days with the corresponding amplitudes 10.10, 12.77 and 3.211. For the Nyquist frequencies the periods are at 258.7 days, 136.1 days and 51.92 days with the corresponding amplitudes 14.01, 10.20 and 5.22. The harmonic series gives periods at 365, 182.5 and 121.6 days with the corresponding amplitudes 9.82, 6.87 and 7.73.

The 50 meters’ population depicts similar periodic lengths in chamber number. The Lomb periodogram gives significant periods at 288 days, 130.9 days and 68.5 days with the corresponding amplitudes 7.39, 10.24 and 5.79. For Nyquist frequencies, the analysis resulted in period lengths of 130.5, 267.4 and 66.9 days with the amplitudes 10.37, 8.20 and 5.22. Here the harmonic series gives periods at 365, 182.5 and 121.6 days with the corresponding amplitudes 6.38, 3.52 and 11.34; note here the very low amplitude of the 180 days period.

**Figure 12:**
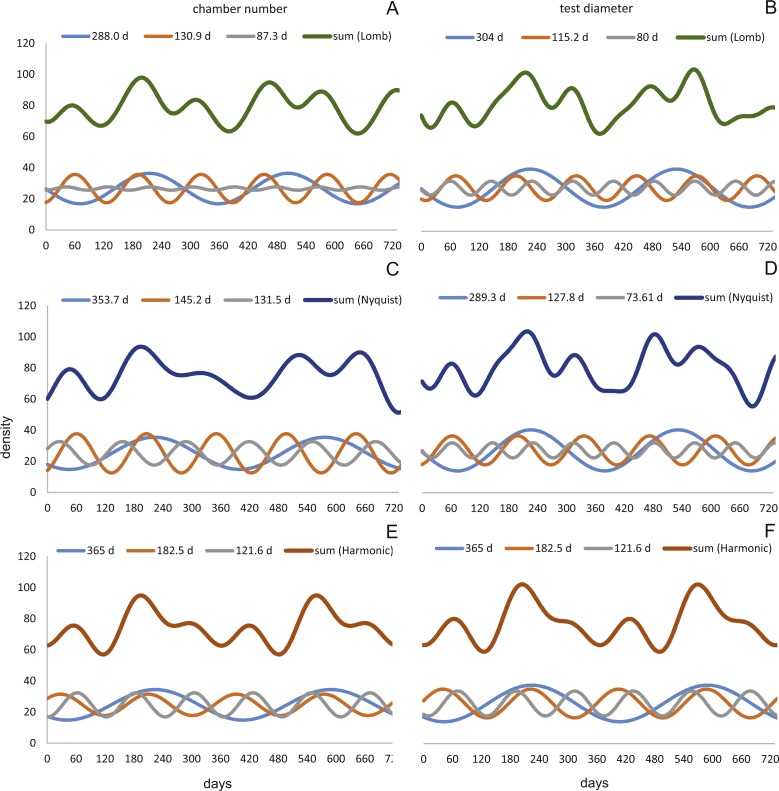
Oscillations in reproduction timing for the 20 meter’s population over 2 years (730 days) illustrating the three most significant sinusoidal functions (sinusoid 1-3) and their sum-of-sinusoids. Based on lomb periodogram (green; based on NoC (A), based on TD (B)), nyquist frequency (blue; based on NoC (C), based on TD (D)) and harmonic series (orange; based on NoC (E), based on TD (F)).

Quite similar periodic lengths can be found based on the inversion of the DIR’s at both water depths, even though the longest period is extended in both populations. The population from 20 m exhibit, according to the Lomb periodogram, periods of 304 days, 125.2 days and 84.7 days with the corresponding amplitudes 12.25, 7.92 and 4.49. For Nyquist frequencies the periods are at 306.3 days, 136.8 days and 84.1 days with the corresponding amplitudes 13.2, 9.31 and 4.88. The harmonic series results in periods of 365, 182.5 and 121.6 days with the amplitudes 11.74, 9.22 and 8.02. This is illustrated in Fig. 12. For further Information on the cycles, see Supplemental Information 2.

**Figure 13:**
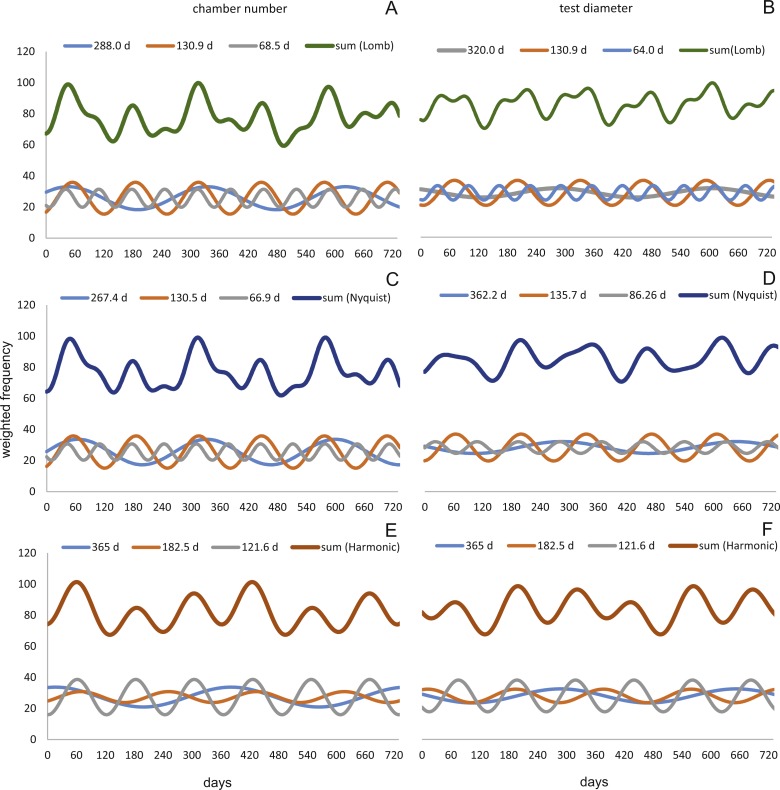
Oscillations in reproduction timing for the 50 meter’s population over 2 years (730 days) illustrating the three most significant sinusoidal functions (sinusoid 1-3) and their sum-of-sinusoids. Based on lomb periodogram (green; based on NoC (A), based on TD (B)), nyquist frequency (blue; based on NoC (C), based on TD (D)) and harmonic series (orange; based on NoC (E), based on TD (F)).

At 50 m water depth the Lomb periodogram gives significant periods at 320 days, 130.9 days and 64 days with corresponding amplitudes 2.99, 8.05 and 4.65. For Nyquist frequencies the analysis resulted in period lengths of 135.7, 86.26 and 362.2 days with amplitudes 8.64, 3.75 and 3.82. Here the harmonic series gives again periods at 365, 182.5, and 121.6 days length, with the amplitudes 4.52, 4.35 and 10.19. As it is illustrated in Fig. 13.

All these oscillations are comparable to those found in the volumetric growth of *H. depressa* (Briguglio & Hohenegger, 2014; Eder, Briguglio & Hohenegger, 2016a; Eder et al., 2016b).

Oscillation around ∼300 and ∼130 days periodic length exhibit amplitudes of similar magnitude at 20 and 50 meters’ populations. However, it can be observed that within the reproductive oscillations at 20 m the long-term cycle (∼300 days) exhibit a higher amplitude, while at 50 m the intermediate cycle (∼130 days) shows the higher amplitude. Oscillations with a periodic length around 180 days can only be found using the harmonic series. They are much more strongly expressed in 20 meter’s population, while at 50 m water depth expression is strongly reduced.

The same patterns with only slightly differences can be observed in the histograms for CBR’s and DIR’s. At 20 m population the reproduction peak in winter ‘14 is weaker and less distinctively expressed in the CBR than in the DIR. Reproduction peaks are equally distinct for CBR and DIR at 50 m, but the histogram for DIR shows a much stronger winter ‘14 peak.

### Life expectancy

The longevity of each specimen of *H. depressa* encompasses the amount of days between sampling date and the estimated birthdate based on individual chamber number and test diameter. Due to different Michaelis–Menten functions for CBR and DIR, the estimated maximal longevity varies. For *H. depressa* from 20 m this is 416 days (NoC) and 482 (TD) and for the 50 meter populations 435 days (NoC) and 480 days in test diameter (TD).

## Discussion

During main reproduction times up to three overlapping generations of *H. depressa* can be detected within one month. By decomposing the population into normally distributed components, chamber-wise and diameter-wise growth within generations can be observed. Both growth models can be computed using either the Michaelis–Menten (MM) function (NoC) or the generalized form of the Michaelis–Menten function (TD). Both result in well fitted averaged chamber building (CBR) and diameter increase rates (DIR). The statistical proven concordance of the function parameters of two generations hint towards the likewise influence of similar seasonal changes. These influence pinpoint towards the most important environmental parameters, which influence LBF distribution: temperature, transparency, hydrodynamics and nutrient content.

The first derivate of the MM indicates the continuously decreasing number of chambers built per day correlated with increasing lifetime. This is very similar for both water depths starting at 20 m with 1.82 chambers/day (Generation 2) and 1.90 chambers/day (Generation 3); at 50 m this is 1.84 chambers/day (Generation 2) and 1.84 chambers/day (Generation 3). Interestingly, the initial growth of *H. depressa* is thus much faster than of *P. venosus* (Kinoshita et al., 2017), which may be caused by the smaller proloculus (∼1/3) and initial chambers size of *H. depressa*. Furthermore, it should be remarked that according to Eder, Hohenegger & Briguglio (2017) only schizonts of *Heterostegina depressa* can be found ∼20 m water depth around Sesoko Jima. Hence, the results of the natural laboratory at the shallower sampling stations are valid only for schizonts. On the contrary, specimens from 50 m are predominantly gamonts, while schizonts are rare (∼ratio 9:1). Therefore, the results of the natural laboratory on the deeper sampling station is valid for gamonts. Chamber building rates between the populations barely differ (see Figs. 7 and  8), which is further indicated by the similar chamber per day rates (see Table 6).

**Table 6:** Summary of chamber building rates and chamber per day rates. CBR building rates and chamber per day rates in daily interval for the first week and afterwards increasing time-intervals.

	20 m		50 m	
Days	NoC	ch per day	NoC	ch per day
1	2	1.8	2	1.8
2	4	1.7	4	1.8
3	6	1.7	5	1.7
4	7	1.6	7	1.6
5	9	1.5	9	1.5
6	10	1.4	10	1.5
7	12	1.4	12	1.4
10	16	1.2	15	1.2
15	21	1.0	21	1.0
30	33	0.6	33	0.6
60	45	0.3	47	0.3
90	52	0.2	54	0.2
120	56	0.1	59	0.1
150	58	0.1	62	0.1
180	60	0.1	64	0.1
210	62	0.0	66	0.1
240	63	0.0	67	0.0
270	64	0.0	68	0.0
300	65	0.0	69	0.0
330	65	0.0	70	0.0
360	66	0.0	71	0.0

Deeper living specimens have a stronger chambers size increase and take more time to build adult-stage chambers Eder, Hohenegger & Briguglio, 2018. This is reflected in a much higher maximal test diameter (9,400 µm as limit for the maximal DIR and 5,846 µm as limit for the mean DIR), while specimens from the shallower sampling stations exhibit a smaller maximal test diameter (6,331 µm as limit for the maximal DIR and 5,049 µm as limit for the mean DIR).

**Figure 14:**
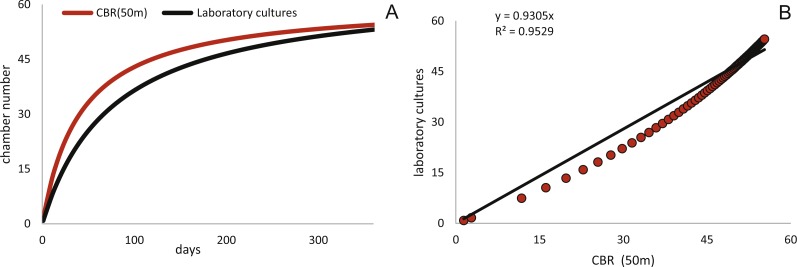
Comparison of the CBR from 50 meter’s population (red) to the observed CBR rates of Röttger (1990). Fitted by a Michaelis-Menten function (black) (A). Correlation between CBR for 50 m population and the CBR of the laboratory cultures (B), illustrating the stronger increase of the CBR gained from the natural laboratory in initial part of the function.

Estimated CBRs gained by the natural laboratory approach exceeds the CBR rates expected from laboratory cultures (Röttger, 1990). The final chamber number is similar in the laboratory cultures, but the CBR function is overall flatter (Fig. 14A). The correlation between the CBRs calculated using the ‘natural laboratory’ and laboratory cultures (Röttger, 1990) illustrates that major differences between the CBR functions are especially strongly expressed during juvenile to early adult stages (Fig. 14B). This accelerated natural growth may be explained by the more favorable conditions in natural habitats (Hohenegger, Briguglio & Eder, 2014).

Schizontic laboratory offspring from even shallower collected environments studied by Röttger, (1972b) result in comparable mean test diameters. The mean test diameter of Röttger’s F_1_ (28.08.1969) reaches 2,184 µm after 253 days, while in the present study a mean test diameter estimated by the fitted function for 20 meter’s population results in 2,332 µm after that time. Final adult size is comparable due to the decreasing growth rate characteristic in adult specimens, therefore the DIR like the CBR becomes much flatter in culture. Cultured individuals only reach ∼1,500 µm after 150 days, while specimens from Sesoko-Jima are already ∼2,000 µm at that time interval. Laboratory cultures of gamonts from Krüger (1994) and Röttger (1990) can be compared with the natural laboratory results from 50 m. Again, a similar pattern is revealed when observing adult test diameters. According to Krüger (1994), gamonts reached 5,000 µm maximal test diameter after 300 days, which fits to the results of the maximal DIR of 50 m of 5,023 µm after 300 days. Surprisingly, the test diameter reported by Röttger (1990) with a mean test diameter of ∼3,610 µm after 186 days exceeds the values achieved by the mean DIR from gamonts ∼2,260 µm. This discrepancy can probably be accredited to the different way of measuring test diameter. In the present study, the maximal test diameter running through the proloculus was measured, which is only possible in thin sections or CT-scans. Further, the comparison of growth of maximal test diameter between laboratory cultures and specimens of the natural habitat is quite problematic due to the continuous test flattening of *H. depressa* with water depth. Within the two studied populations, the degree of flattening is constant, since monthly samples originate from more or less similar water depths. However, if these shape changes are due to an ecological imprint or epigenetically inherited is yet unclear, while recent work on *Operculina* hints towards the later (Oron et al., 2018). Hence the ambient light conditions of the cultures, as well as the spectra of the chosen illuminant might hamper the comparability of DIRs. Generally, the high ecomorphological variability of *H. depressa* (Eder et al., 2016b), whose influencing factors are not fully understood, indicates that chamber number and chamber volume are better suited as indicators for quantification of growth than test diameter. Hence, differences between CBRs from laboratory cultures and natural population underline the opinion of Hohenegger, Briguglio & Eder (2014) that culture methods for larger benthic foraminifera still need to be improved before growth observations in the tank can be directly related to field observations. Further, Wöger et al. (2016) commented on the high mortality in laboratory cultures and on the higher amount of so-called Kummerkammern (sensu Hottinger & Scheuring, 1997), that are built by laboratory specimens.

Even though slightly different frequency/density histograms exist using birth dates of specimens based on CBR and DIR, both indicate a continuous reproduction with two peaks throughout the year, which explains the presence of differently sized megalospheric generations within the studied monthly samples. The unequal distances between winter reproductions as seen in Figs. 10 and 11 can be explained by the extremely low number of *H. depressa* from April samples in contrast to *P. venosus*, due to the patchy distribution of LBF around Sesoko Jima (Kinoshita et al., 2017). Interestingly, this is in stark contrast to former reproduction studies on other LBF done around Okinawa. On the one hand, porcelainous species (e.g., *Peneroplis antillarum* in Hohenegger, 2006; Hohenegger, Briguglio & Eder, 2014; *Amphisorus hemprichii* in Zohary, Reiss & Hottinger, 1980) and hyaline species (e.g., *Calcarina gaudichaudi* in Hohenegger, 2006*; Baculogypsina sphaerulata* in Sakai & Nishihira, 1981; Hohenegger, 2006) studied in the subtropics showed a single mass reproduction restricted to June, while porcelaneous *Amphisorus kudakajimaeinsis* on the other hand exhibits two events restricted to June and November (Fujita, Nishi & Saito, 2000; Hohenegger, 2006; Hohenegger, Briguglio & Eder, 2014; Zohary, Reiss & Hottinger, 1980). A third reproduction mode has been observed in tropical eulittroal *B. sphaerulata*, which shows constant birth rates over the year lacking major reproduction peaks (Fujita et al., 2016), which seems to be characteristic for tropical LBFs.

**Figure 15:**
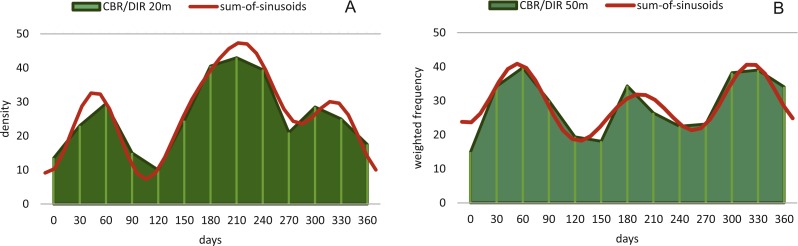
Illustration of histograms for estimated birthdates of all investigated specimens using combined densities of CBR and DIR. The histogram of 20 meter’s population is given in simple counts (A). The histogram of 50 meter’s population is given in weighted frequencies (B). Both are fitted as sum-of-sinusoids illustrating the overall trend.

Reproduction events in *Heterostegina depressa* and the influencing environmental parameters can be addressed in more detail using frequency/density diagrams of estimated birthdates (see Figs. 10 and 11). CBR and DIR differ from each other slightly since CBR and DIR do not correlate linearly. NoC increases much faster than TD, especially in the post-embryonic and juvenile state. In late adult stages, TD increases much faster, and continues till it reaches the maximum (see Fig. 9). Regardless, major reproduction peaks throughout the year do not change between histograms for CBR and DIR (Figs. 10 and 11), hence these trends can be significantly fitted to a combined diagram using CBR and DIR by sum-of-sinusoids (Fig. 15).

**Figure 16:**
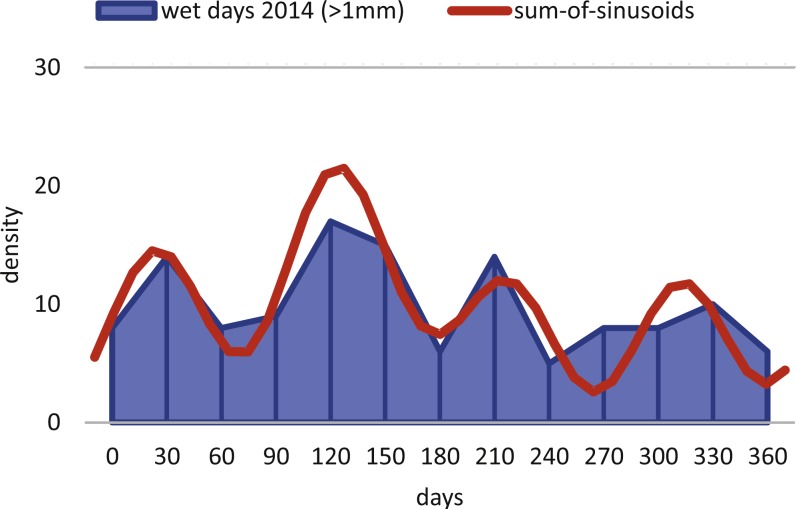
Precipitation. Histogram of the monthly wet days (>1 mm precipitation) of the sampling area (Nago City, Okinawa) for the sampling year 2014.

Within *H. depressa* of different water depths a mixture of the aforementioned three different reproduction modes can be observed. While CBR and DIR histograms (Figs. 10 and 11) from both water depths express peak events and a continuous reproduction throughout the year, the reproduction event in summer is dominant at 20 m, characterized by the highest peak 3-times stronger than in winter reproduction peaks. This means that for *Heterostegina depressa* schizonts a reproduction strategy with a combination of all three reproductive modes can be assumed: subtropical with one peak (dominance in summer), subtropical with two peaks (winter reproduction) and tropical without peaks (background reproduction). In schizonts reproductions follows a relative simple asexual mode, since they keep ‘cloning’ themselves by apogamic fission with exclusion of agamonts Röttger, Krüger & De Rijk (1990). It is probably initiated by the approaching monsoon front and reaches its peak when water temperature and salinity are the highest (Hohenegger, 2004). The dominant summer peak in *H. depressa* schizonts is in concordance with the assumption by Lipps (1982) that schizogeny dominates during stress conditions. The much weaker winter peaks can be correlated with a maximum amount of wet days (>1 mm precipitation) and low temperatures in winter (based on the records of the Japanese meteorological agency). The timing of winter peaks is reflected in the amount of wet days (Fig. 16), meaning, that the unequal timing might rather be meteorically influenced than by missing samples.

In the 50 m population this strong dominance of summer reproduction is lost and the pattern is reversed. In the density histogram for CBRs (Fig. 14B) a smaller peak during summer is barely recorded, while winter reproduction is much more expressed. This is relativized when using combined DIR/CBR diagrams (Fig. 15B): Here summer peaks are only slightly less pronounced. Therefore, *H. depressa* gamonts show a reproduction strategy combining two reproductive modes, subtropical with two peaks (∼equally high summer and winter events) and tropical without peaks (background reproduction). The interpretation of reproduction peaks in gamonts is more complex. This other part of the trimorphic lifecycle depends on the constant alternation of gamonts and agamonts. However, due to the low ratio of agamonts to gamonts, agamonts are hardly sampled in a sufficient amount to infer peak abundances throughout the year. Main events of gamete expulsion can still be inferred from peak abundances of gamonts. Meaning, that the onset of gamete production coincides with the decline in abundance of the largest gamont specimens (>60 chambers) (see Fig. 4, May–June ’14 and April–May ’15) and continues into the early winter months where no large gamonts are present anymore. Due to the higher number of chambers in microspheres as in megalospheres (roughly two times) the estimated life expectancy of ∼3 years by Briguglio & Hohenegger (2014) seems sensible. Based on this assumption and a yearly production of gametes, agamonts should theoretically be present consistently throughout the year with preferred reproduction mainly during winter and less during summer. However, the environmental influence is less clear, since most environmental parameters are dampened due to the greater water depth. Strong fluctuations in water temperature and salinity do not reach down to 50 m water depth. Only periods of increased precipitation (e.g., during the passing of the monsoon front) might reach deeper water layers, reducing transparency and nutrient input Wöger et al. (2016) and such events may trigger agamont reproduction.

Maximum longevity of *Heterostegina depressa* from ∼20 m water depth (schizonts) is estimated based on CBR to be 416 days and based on DIR 482 days. For the population from 50 m, which are interpreted at gamonts, the maximal estimated longevity is 435 days (CBR) and 480 days (DIR). The 416 days of life expectancy in schizonts correlates with results from laboratory cultures by Röttger (1972b), where schizonts lived up to a maximum of 13 months and 1 day (∼390 days). For gamonts, the maximal observed longevity in cultures (Krüger, 1994) is much lower at 10 months (300 days). However, this short life time expectancy would need a much higher growth rate than has been calculated for gamonts to reach comparable maximal chamber numbers as observed in the field. Hence, the assumption seems to be reasonable that the maximum longevity of *H. depressa* could be around one and a half years, comparable to the results of the ‘natural laboratory’ applied on *P. venosus* (Kinoshita et al., 2017). This expectancy is quite similar to some eulittoral LBF species, e.g., *Calcarina gaudichaudi* and *B. sphaerulata* (Hohenegger, 2006). The stronger difference between the two estimates for the shallower population is due to a much stronger deviation from a linear correlation of CBR and DIR. The reason for this is the more drastic transition from involute to evolute growth in 20 meter’s specimens, resulting in a drastic change in the DIR at late growth stages. The degree of test flattening could not be connected to proloculus size, which is the main morphological differentiator between schizonts and gamonts (Eder, Hohenegger & Briguglio, 2018).

## Conclusion

Based on population dynamic studies of megalospheric *H. depressa* from ∼20 and ∼50 water depth, an averaged chamber building rate (CBR) and test diameter increase rate (DIR) have been estimated. Since in Sesoko-Jima only schizonts are represented at 20 m and gamonts dominate at 50 m water depth, the results of the natural laboratory from different water depths can be applied either for schizonts (20 meter samples) or gamonts (50 meter samples). While the CBR and the maximal chamber number between the two generations do not differ significantly, the DIR including the maximal test diameter greatly deviate between the two populations. This differences in DIR, however, are due to the strong test flattening at 50 m rather than different growth between the two megalospheric generations. Maximal life expectancy of both megalospheric generations can be assumed to be around 1.5 years, similar to *P. venosus*, *B. sphaerulata* and *C. gaudichaudi* (Hohenegger, 2006; Kinoshita et al., 2017; Sakai & Nishihira, 1981). A similar prediction for agamonts of *H. depressa* was not possible due to the few number of agamonts found during the presented study. However, based on the amount of chambers in the largest agamonts, which is roughly twice as high as in megalospheric forms (∼130), the estimation of 3 years by Briguglio & Hohenegger (2014) seems legitimate.

Summarizing the reproduction of *H. depressa*, schizogeny occurs continuously throughout the year, with the highest reproduction peak during the summer months (July–August) and weaker reproduction during winter months (November–December; February–March). The summer peak coincides with the strongest environmental disturbances of the habitat. It is probably initiated by the passing of the monsoon front in May and peaks when water temperature and salinity are the highest. The reproduction timing of gamonts also occurs continuously over the year with summer (July) and winter (November–December; February–March) peaks. In contrast to schizonts, summer peaks are strongly reduced and more weakly expressed than winter reproduction, even though most environmental parameters are dampened due to the greater water depth. Reproduction in *H. depressa* schizonts shows a combination of reproduction modes observed in other LBF. It expresses two reproduction peaks per year (*A. kudakajimaensis*; peaks in June and November), with a dominance towards the summer peak (*A. hemprichii*, mass reproduction in summer) and continuous background reproduction (tropical *B. sphaerulata*; continuous reproduction). Gamonts of *H. depressa* do not express the dominance of a summer peak and show, similar to *A. kudakajimaensis* , two peaks per year overlying a continuous reproduction like tropical *B. sphaerulata*. Earlier studies on *B. sphaerulata* showed that populations in subtropical regions rather limit their reproduction from late spring to early summer. Further studies on the reproduction timing of *H. depressa* should consider the bathymetric change presented in this study. In addition, it is interesting in which way reproductive peaks depend on external influence (e.g., precipitation) and the question arises if the macro weather situation in the subtropic and tropic pacific influences reproduction of larger foraminifera.

The successful application of the ‘natural laboratory approach’ exemplifies that this methodology can be used for other larger foraminifera and shallow marine organism, where size can be quantified in monthly samples (e.g., scleractinians, molluscs and brachiopods).

##  Supplemental Information

10.7717/peerj.6096/supp-1 Supplemental Information 1 Test for equal means Student’s t tests were used to check the coincidence in parameters for CBRs and DIRs for 20 m and 50 m samples.

10.7717/peerj.6096/supp-2 Supplemental Information 2 Summary for reproductive cycles Summarizing parameters and significance for all calculated cycles.

10.7717/peerj.6096/supp-3 Supplemental Information 3 Raw data 20 m Chamber number and test diameter (µm) for each specimen for each sampling.

10.7717/peerj.6096/supp-4 Supplemental Information 4 Raw data 50 m Chamber number and test diameter (µm) for each specimen for each sampling.

## Additional Information and Declarations

### Data Availability

The following information was supplied regarding data availability: The raw data are provided in the Supplemental Files. The primary data is available at: Wolfgang Eder, Julia Woeger, Shunichi Kinoshita, Johann Hohenegger, & Antonino Briguglio. (2018). Growth estimation of the larger foraminifer Heterostegina depressa by means of population dynamics (3D datasets)). Zenodo. http://doi.org/10.5281/zenodo.1477635.

## References

[ref-1] Banner FT, Hodgkinson RL (1991). A revision of the foraminiferal subfamiliy Heterostegininae. Revista Espanola de Micropaleontologia.

[ref-2] Baumgarten S, Laudien J, Jantzen C, Häussermann V, Försterra G (2014). Population structure, growth and production of a recent brachiopod from the Chilean fjord region. Marine Ecology.

[ref-3] Beavington-Penney SJ, Racey A (2004). Ecology of extant nummulitids and other larger benthic foraminifera: applications in palaeoenvironmental analysis. Earth-Science Reviews.

[ref-4] Biekart J, Bor T, Röttger R, Drooger C, Meulenkamp J (1985). *Megalospheric Heterostegina* depressa from Hawaii in sediments and laboratory cultures.

[ref-5] Briguglio A, Hohenegger J (2009). Nummulitid hydrodynamics: an example using *Nummulites globulus* Leymerie. Bollettino Della Societa Paleontologica Italiana.

[ref-6] Briguglio A, Hohenegger J (2014). Growth oscillation in larger foraminifera. Paleobiology.

[ref-7] Briguglio A, Kinoshita S, Wolfgring E, Hohenegger J (2016). Morphological variations in *Cycloclypeus carpenteri*: multiple embryos and multiple equatorial layers. Palaeontologia Electronica.

[ref-8] Cosovic V, Drobne K, Moro A (2004). Paleoenvironmental model for Eocene foraminiferal limestones of the Adriatic carbonate platform (Istrian Peninsula). Facies.

[ref-9] Eder W, Briguglio A, Hohenegger J (2016a). Growth of *Heterostegina depressa* under natural and laboratory conditions. Marine Micropaleontology.

[ref-10] Eder W, Hohenegger J, Briguglio A (2017). Depth-related morphoclines of megalospheric test of *Heterostegina depressa* d’Orbigny: biostratigraphic and palaeobiological implications. PALAIOS.

[ref-11] Eder W, Hohenegger J, Briguglio A (2018). Test flattening in the larger foraminifer *Heterostegina depressa*: predicting bathymetry from axial sections. Paleobiology.

[ref-12] Eder W, Hohenegger J, Torres-Silva AI, Briguglio A (2016b). Morphometry of the larger foraminifera *Heterostegina* explaining environmental dependence, evolution and paleogeographic diversification.

[ref-13] Ferrandez-Canadell C, Briguglio A, Hohenegger J, Woger J (2014). Test fusion in adult foraminifera: a review with new observations of an Early Eocene *Nummulites* specimen. Journal of Foraminiferal Research.

[ref-14] Fujita K, Nishi H, Saito T (2000). Population dynamics of Marginopora kudakajimensis Gudmundsson (Foraminifera: Soritidae) in the Ryukyu Islands, the subtropical northwest Pacific. Marine Micropaleontology.

[ref-15] Fujita K, Otomaru M, Lopati P, Hosono T, Kayanne H (2016). Shell productivity of the large benthic foraminifer Baculogypsina sphaerulata, based on the population dynamics in a tropical reef environment. Coral Reefs.

[ref-16] Hallock P (1985). Why are larger foraminifera large?. Paleobiology.

[ref-17] Hallock P (1988). The role of nutrient availability in bioerosion: consequences to carbonate buildups. Palaeogeography, Palaeoclimatology, Palaeoecology.

[ref-18] Hallock P, Forward LB, Hansen HJ (1986). Influence of environment on the test shape of *Amphistegina*. Journal of Foraminiferal Research.

[ref-19] Hallock P, Röttger R, Wetmore K, Lee JJ, Roger Anderson O (1991). Hypotheses on form and function in foraminifera. Biology of Foraminifera.

[ref-20] Hammer Ø, Harper D, Ryan P (2001). PAST: paleontological statistics software package for education and data analysis. Palaeontologia Electronica.

[ref-21] Hohenegger J (2000). Coenoclines of larger foraminifera. Micropaleontology.

[ref-22] Hohenegger J (2004). Depth coenoclines and environmental considerations of western Pacific larger foraminifera. The Journal of Foraminiferal Research.

[ref-23] Hohenegger J (2006). The importance of symbiont-bearing benthic foraminifera for West Pacific carbonate beach environments. Marine Micropaleontology.

[ref-24] Hohenegger J (2009). Functional shell geometry of symbiont-bearing benthic foraminifera. Galaxea.

[ref-25] Hohenegger J (2011). Growth-invariant meristic characters. Tools to reveal phylogenetic relationships in Nummulitidae (Foraminifera). Turkish Journal of Earth Sciences.

[ref-26] Hohenegger J (2018). Foraminiferal growth and test development. Earth-Science Reviews.

[ref-27] Hohenegger J, Briguglio A, Eder W, Kitazato H, Bernhard J (2014). The natural laboratory of symbiont bearing benthic foraminifera: studying individual growth and population dynamics under natural conditions. Experimental approaches in foraminifera: collection, maintenance and experiments.

[ref-28] Hohenegger J, Yordanova E, Hatta A (2000). Remarks on West Pacific Nummulitidae (foraminifera). Journal of Foraminiferal Research.

[ref-29] Hohenegger J, Yordanova E, Nakano Y, Tatzreiter F (1999). Habitats of larger foraminifera on the upper reef slope of Sesoko Island, Okinawa, Japan. Marine Micropaleontology.

[ref-30] Hottinger L (1982). Larger foraminifera, giant cells with a historical background. Naturwissenschaften.

[ref-31] Hottinger L (2006b). The depth-depending ornamentation of some lamellar-perforate foraminifera. Symbiosis.

[ref-32] Hottinger L, Scheuring V (1997). Glossary of terms used in foraminiferal research.

[ref-33] Kinoshita S, Eder W, Wöger J, Hohenegger J, Briguglio A (2017). Growth, chamber building rate and reproduction time of *Palaeonummulites venosus* (Foraminifera) under natural conditions. Coral Reefs.

[ref-34] Krüger R (1994). Untersuchungen zum Entwicklungsgang rezenter Nummulitiden: heterostegina depressa, Nummulites venosus und Cycloclypeus carpenteri. PhD thesis.

[ref-35] Larsen A, Drooger C (1977). Relative thickness of the test in the *Amphistegina* species of the Gulf of Elat. Utrecht Micropaleontological Bulletin.

[ref-36] Lee J, McEnery M, Shilo M, Reiss Z (1979). Isolation and cultivation of diatom symbionts from larger Foraminifera (Protozoa). Nature.

[ref-37] Leutenegger S (1977). Reproduction cycles of larger foraminifera and depth distribution of generations. Utrecht Micropaleontological Bulletins.

[ref-38] Lietz R (1996). Untersuchungen zur Individualentwicklung der Großforaminifere Cycloclypeus carpenteri Carpenter (1856). Master’s thesis.

[ref-39] Lipps J (1982). Biology/paleobiology of foraminifera. Studies in geology, notes for a short course 6 (Foraminifera).

[ref-40] Lopez S, France J, Gerrits W, Dhanoa M, Humphries D, Dijkstra J (2000). A generalized Michaelis–Menten equation for the analysis of growth. Journal of Animal Science.

[ref-41] Michaelis L, Menten M (1913). Die Kinetik der Invertinwirkung. Biochemische Zeitschrift.

[ref-42] Muller PH (1974). Sediment production and population biology of the benthic foraminifer *Amphistegina madagascariensis*. Limnology and Oceanography.

[ref-43] Nobes K, Uthicke S, Henderson R (2008). Is light the limiting factor for the distribution of benthic symbiont bearing foraminifera on the Great Barrier Reef?. Journal of Experimental Marine Biology and Ecology.

[ref-44] Oron S, Abramovich S, Almogi-Labin S, Woeger J, Erez J (2018). Depth related adaptions in symbiont bearing benthic foraminifera: new insights from a filed experiment on *Operculina amoonoides*. Scienitific Reports.

[ref-45] Press W, Teukolsky S, Vetterling W, Flannery B (1992). Numerical recipes. The art of scientific computing.

[ref-46] Renema W (2005). Depth estimation using diameter-thickness ratios in larger benthic foraminifera. Lethaia.

[ref-47] Renema W, Cotton L (2015). Three dimensional reconstructions of Nummulites tests reveal complex chaber shapes. PeerJ.

[ref-48] Röttger R (1972a). Analyse von Wachstumskurven von *Heterostegina depressa* (Foraminifera: Nummulitidae). Marine Biology.

[ref-49] Röttger R (1972b). Die Kultur von *Heterostegina depressa* (Foraminifera: Nummulitidae). Marine Biology.

[ref-50] Röttger R (1974). Larger foraminifera: reproduction and early stages of development in *Heterostegina depressa*. Marine Biology.

[ref-51] Röttger R, Takanayagi Y, Saito T (1990). Biology of Larger Foraminifera: status of the hypothesis of Trimorphism and Ontogeny of the Gamont of Heterostegina depressa. Studies in Benthic Foraminifera.

[ref-52] Röttger R, Krüger R, De Rijk S (1990). Trimorphism in foraminifera (Protozoa)—verification of an old hypothesis. European Journal of Protistology.

[ref-53] Sakai K, Nishihira M (1981). Population study of the benthic foraminifer Baculogypsina sphaerulata on the Okinawan reef flat and preliminary estimation of its annual production.

[ref-54] Schmidt D, Rayfield E, Cocking A, Marone F (2013). Linking evolution and development: synchrotron Radiation X-ray tomographic microscopy of planktic foraminifers. Palaeontology.

[ref-55] Shannon CE (1949). Communication theory of secrecy systems. Bell System Technical Journal.

[ref-56] Speijer RP, Van Loo D, Masschaele B, Vlassenbroeck J, Cnudde V, Jacobs P (2008). Quantifying foraminiferal growth with high-resolution X-ray computed tomography: new opportunities in foraminiferal ontogeny, phylogeny, and paleoceanographic applications. Geosphere.

[ref-57] Ujiié H, Shioya F (1980). Sediment in the Bay of Nago and around the island of Sesoko, Okinawa. Sesoko Marine Science Laboratory, Technical Reports.

[ref-58] Uthicke S, Nobes K (2008). Benthic Foraminifera as ecological indicators for water quality on the Great Barrier Reef. Estuarine, Coastal and Shelf Science.

[ref-59] Wöger J, Briguglio A, Eder W, Kinoshita S, Hohenegger J (2016). First results of a long-term cultivation experiment of different species of Nummulitidae (Foraminifera) from the island of Sesoko (Okinawa, Japan).

[ref-60] Yordanova EK, Hohenegger J (2007). Studies on settling, traction and entrainment of larger benthic foraminiferal tests: implications for accumulation in shallow marine sediments. Sedimentology.

[ref-61] Zohary T, Reiss Z, Hottinger L (1980). Population dynamics of Amphisorus hemprichii (Foraminifera) in the Gulf of Elat (Aqaba), Red Sea. Eclogae Geologicae Helvetiae.

